# Network Pharmacology and Experimental Verification to Unveil the Mechanism of N-Methyl-D-Aspartic Acid Rescue Humantenirine-Induced Excitotoxicity

**DOI:** 10.3390/metabo13020195

**Published:** 2023-01-28

**Authors:** Xue-Jia Qi, Chong-Yin Huang, Meng-Ting Zuo, Meng-Die Gong, Si-Juan Huang, Mo-Huan Tang, Zhao-Ying Liu

**Affiliations:** 1 College of Veterinary Medicine, Hunan Agricultural University, Changsha 410128, China; 2 Hunan Engineering Technology Research Center of Veterinary Drugs, Hunan Agricultural University, Changsha 410128, China

**Keywords:** humantenirine, excitotoxicity, NMDA, NMDAR, network pharmacology, *Gelsemium*

## Abstract

*Gelsemium* is a medicinal plant that has been used to treat various diseases, but it is also well-known for its high toxicity. Complex alkaloids are considered the main poisonous components in *Gelsemium*. However, the toxic mechanism of *Gelsemium* remains ambiguous. In this work, network pharmacology and experimental verification were combined to systematically explore the specific mechanism of *Gelsemium* toxicity. The alkaloid compounds and candidate targets of *Gelsemium*, as well as related targets of excitotoxicity, were collected from public databases. The crucial targets were determined by constructing a protein–protein interaction (PPI) network. Subsequently, Gene Ontology (GO) and the Kyoto Encyclopedia of Genes and Genomes (KEGG) were used to explore the bioprocesses and signaling pathways involved in the excitotoxicity corresponding to alkaloids in *Gelsemium*. Then, the binding affinity between the main poisonous alkaloids and key targets was verified by molecular docking. Finally, animal experiments were conducted to further evaluate the potential mechanisms of *Gelsemium* toxicity. A total of 85 alkaloids in *Gelsemium* associated with 214 excitotoxicity-related targets were predicted by network pharmacology. Functional analysis showed that the toxicity of *Gelsemium* was mainly related to the protein phosphorylation reaction and plasma membrane function. There were also 164 pathways involved in the toxic mechanism, such as the calcium signaling pathway and MAPK signaling pathway. Molecular docking showed that alkaloids have high affinity with core targets, including MAPK3, SRC, MAPK1, NMDAR_2B_ and NMDAR_2A_. In addition, the difference of binding affinity may be the basis of toxicity differences among different alkaloids. Humantenirine showed significant sex differences, and the LD_50_ values of female and male mice were 0.071 mg·kg^−1^ and 0.149 mg·kg^−1^, respectively. Furthermore, we found that N-methyl-D-aspartic acid (NMDA), a specific NMDA receptor agonist, could significantly increase the survival rate of acute humantenirine-poisoned mice. The results also show that humantenirine could upregulate the phosphorylation level of MAPK3/1 and decrease ATP content and mitochondrial membrane potential in hippocampal tissue, while NMDA could rescue humantenirine-induced excitotoxicity by restoring the function of mitochondria. This study revealed the toxic components and potential toxic mechanism of *Gelsemium*. These findings provide a theoretical basis for further study of the toxic mechanism of *Gelsemium* and potential therapeutic strategies for *Gelsemium* poisoning.

## 1. Introduction

*Gelsemium*, a genus of the Loganiaceae family, comprises three species: the Asian *Gelsemium elegans* (Gardner and Chapm.) Benth. and two North American related species, *Gelsemium sempervirens* (L.) J.St.-Hil. and *Gelsemium rankinii* Small [1]. *Gelsemium elegans*, as a traditional Chinese medicine, has been used to treat skin disorders, malignant tumors and pain for a long time [2,3]. *Gelsemium sempervirens* is used in homeopathy to treat anxiety, neuralgia, migraine and spastic diseases [4,5]. Due to their variety and high biological activity, alkaloids are considered to be the main active substances in *Gelsemium*. According to the characteristics of chemical structure, alkaloids can be divided into six types: the gelsedine type, gelsemine type, humantenine type, koumine type, sarpagine type and yohimbane type [3,5]. The toxicity of different alkaloids is greatly different, and gelsedine-type and humantenine-type alkaloids are the most toxic in *Gelsemium*. The LD_50_ of these two types of alkaloids for intraperitoneal injection in mice are usually lower than 1 mg·kg^−1^ [6,7]. There are many cases of *Gelsemium* poisoning, some of which have even led to death [8,9], which seriously limits its application.

Mechanistic studies for *Gelsemium* toxicity are scarce at present. A few studies have reported that the toxicity of *Gelsemium* is closely related to gamma-aminobutyric acid receptor (GABAR) [10,11] and glycine receptors [12]. Recently, a phosphoproteomics study revealed that an N-methyl-D-aspartic acid receptor (NMDAR)-mediated excitotoxicity signaling pathway is linked to the death of gelsenicine (one of the toxic alkaloids in *Gelsemium*) poisoning [13]. Therefore, it is hypothesized that the toxicity of *Gelsemium* is associated with excitotoxicity, which is consistent with the typical symptoms of *Gelsemium* poisoning, including dyspnea and convulsions. However, most of the existing studies on the toxicity of *Gelsemium* are still imperfect and the specific reasons for the toxicity differences among different alkaloids are still unclear.

Network pharmacology [14], a burgeoning interdisciplinary subject, highlights comprehensive thinking, focuses on the interaction among drugs, targets and diseases, and takes advantage of various means and technologies, such as molecular docking [15] and enrichment analysis [16], to screen the active ingredients, explore the potential core targets, and reveal the mechanisms of drugs. Network pharmacology is widely used in research to reveal the molecular mechanism of various drugs and poisons [17], and the reliability and accuracy of the technical methods used have been recognized by international standards. In the present study, we used the network pharmacology method to find the possible targets of *Gelsemium* toxicity, and used the molecular docking method to verify the binding affinity of *Gelsemium* alkaloids with these core targets. Finally, an acute poisoning model of humantenirine in mice was established to further reveal the potential toxic mechanism of *Gelsemium*. This study is expected to lay a theoretical basis for the development and utilization of *Gelsemium*.

## 2. Materials and Methods

### 2.1. Collection and Screening of Gelsemium Alkaloids

The alkaloid compounds in *Gelsemium* were obtained by referring to the literature [3,5] and searching public databases, including the Traditional Chinese Medicines Integrated Database (TCMID, http://119.3.41.228:8000/tcmid/, accessed on 20 July 2022) [18] and the Traditional Chinese Medicine Database @ Taiwan (TCMTW, http://tcm.cmu.edu.tw/zh-tw/, accessed on 20 July 2022) [19]. According to the Drug likeness parameters of the SwissADME platform(http://www.swissadme.ch/index.php, accessed on 22 July 2022) [20], the alkaloids that have bioavailability scores ≥ 30% and meet at least two of the Lipinski rules (Lipinski, Ghost, Veber, Egan, and Muegge) were screened. The qualified alkaloids were finally determined to be candidate alkaloid components in *Gelsemium*.

### 2.2. Screening of Targets Corresponding to the Alkaloid Components

The canonical SMILES of alkaloid components were retrieved from the open database PubChem (https://pubchem.ncbi.nlm.nih.gov, accessed on 25 July 2022). Canonical SMILES were imported into the SwissTargetPrediction webtool (http://www.swisstargetprediction.ch, accessed on 25 July 2022) [21] to estimate the targets corresponding to each alkaloid in Homo sapiens. The targets with probability ≥0.1 were selected as potential targets. For the components not included in the SwissTargetPrediction database, the BATMAN-TCM online analysis tool (http://bionet.ncpsb.org.cn/batman-tcm/index.php/Home/Index/index, accessed on 25 July 2022) was used as a supplement. The potential targets of alkaloids were obtained by taking a score cutoff of ≥ 10 and P ≤ 0.05 as screening conditions. The components without target information in both databases were excluded. The targets of all compounds were combined, and then the repeated targets were removed to obtain the targets corresponding to the alkaloid components of *Gelsemium*.

### 2.3. Identification of Targets Related to Excitotoxicity

Targets for “excitotoxicity” were obtained from the GeneCards database (https://www.genecards.org/, accessed on 28 July 2022) [22] and National Center for Biotechnology Information databases (NCBI, https://www.ncbi.nlm.nih.gov/, accessed on 28 July 2022). The obtained targets were summarized, and then the repetitive targets were eliminated to acquire the targets related to excitotoxicity.

### 2.4. Prediction of Targets of Alkaloid Components Associated with Excitotoxicity

The intersection between the targets related to the alkaloid components of *Gelsemium* and excitotoxicity-associated targets was visualized by Venny 2.1 (https://bioinfogp.cnb.csic.es/tools/venny/index.html, accessed on 02 August 2022) [23].

### 2.5. Protein-Protein Interaction (PPI) Network Construction and Analysis

The targets of intersection were submitted to the STRING database (https://string-db.org/cgi/input.pl, accessed on 02 August 2022) [24] to construct the PPI network. The interaction score was set to 0.7, which indicates high confidence. In addition, the species was restricted to “*Homo sapiens*”. The result was saved as a “tsv” file. Finally, the results were input into Cytoscape 3.6.0 software to analyze core targets according to the Degree, ClosenessCentrality and BetweennessCentrality, which were used to evaluate the topological importance of nodes in the network [25].

### 2.6. Gene Ontology (GO) and Kyoto Encyclopedia Genes Genomes (KEGG) Pathway Enrichment Analysis

GO and KEGG pathway enrichment analyses were carried out to explore the bioprocesses and signaling pathways involved in the excitotoxicity corresponding to alkaloids in *Gelsemium*. These targets were input into the Database for Annotation, Visualization and Integrated Discovery (DAVID, https://david.ncifcrf.gov/, accessed on 02 August 2022) [26]. Then, the results of the enriched GO terms, including biological process (BP), cellular component (CC) and molecular function (MF) were visualized by the bioinformatics (http://www.bioinformatics.com.cn, accessed on 02 August 2022), as well as the dot bubble chart of KEGG pathway enrichment.

### 2.7. Construction of the Alkaloid–Target–Pathway Network

A compound–target network and a target–pathway network were constructed by using Cytoscape v3.6.0 software. In the network, different types of nodes represent alkaloid components, targets and pathways. The connection between nodes indicates the interactions between components and targets or between targets and pathways. Three topological characteristic parameters (Degree, ClosenessCentrality and BetweennessCentrality) were used to identify the main poisonous components in *Gelsemium*.

### 2.8. Molecular Docking

The crystal structures of MAPK3, SRC, MAPK1, NMDAR_2B_ and NMDAR_2A_ were obtained from the RCSB Protein Data Bank (https://www.rcsb.org/, accessed on 18 August 2022, PDB codes: 4QTB, 2H8H, 4QTA, 7EU8 and 7EU7). All original ligands (including (3R)-1-(2-oxo-2-{4-[4-(pyrimidin-2-yl)phenyl]piperazin-1-yl}ethyl)-N-[3-(pyridin-4-yl)-2H-indazol-5-yl]pyrrolidine-3-carboxamide, N-(5-CHLORO-1,3-BENZODIOXOL-4-YL)-7-[2-(4-METHYLPIPERAZIN-1-YL)ETHOXY]-5-(TETRAHYDRO-2H-PYRAN-4-YLOXY)QUINAZOLIN-4-AMINE, (3R)-1-(2-oxo-2-{4-[4-(pyrimidin-2-yl)phenyl]piperazin-1-yl}ethyl)-N-[3-(pyridin-4-yl)-2H-indazol-5-yl]pyrrolidine-3-carboxamide, and S-ketamine) and water molecules were removed, and hydrogen atoms and charges were added to the macromolecules. The three-dimensional structures of *Gelsemium* alkaloids downloaded from the PubChem database and optimized by Chem 3D Pro15.0 were used as the ligand. Molecular docking was finalized by AutoDock Vina [27]. The size of the gridbox was fixed to 40 × 40 × 40 angstroms, with a spacing of 0.375 angstrom. All the parameters of the genetic algorithm (GA) were set to the default values. The conformers with the lowest binding energy were selected for analysis.

### 2.9. Animal Experiments

#### 2.9.1. The LD_50_ of Acute Humantenirine Poisoning

Humantenirine was obtained from Chengdu Man Si Te Biotechnology Co., Ltd. (Chengdu, China) with a batch number of MUST-21052807 and purity of 98.4%. ICR mice (18–22 g) were provided by Hunan SJA Laboratory Animal Co., Ltd. (Changsha, China). The mice were reared in a standard facility. The animal experiments were approved by the Ethics Committee of Hunan Agricultural University (batch number 2020–43). The mice were randomly divided into 5 female groups and 5 male groups (n = 5). Humantenirine was injected into female mice intraperitoneally at 0.045, 0.056, 0.069, 0.086 and 0.11 mg·kg^−1^. Male mice were given humantenirine by intraperitoneal injection at doses of 0.1, 0.12, 0.13, 0.16 and 0.18 mg·kg^−1^. After administration, the poisoning symptoms and mortality were observed for 14 consecutive days. Finally, the lethal dose (LD_50_) was assessed by the Bliss method.

#### 2.9.2. The Antidotic Effect of NMDA on Humantenirine Poisoning

A total of 20 female mice were randomly assigned to two groups: the control group and the NMDA group (n = 10). In the control group, humantenirine, at a dose of 0.11 mg·kg^−1^ according to the 100% lethal dose we explored earlier, was injected intraperitoneally into mice. The mice in the NMDA group were injected with 25 mg·kg^−1^ NMDA intraperitoneally 20 min before humantenirine injection. The dose of NMDA used in the experiment was based on our previous exploration. Then, the death of the mice in the two groups was recorded.

#### 2.9.3. Drug Treatment and Sample Collection

Female mice were randomly assigned to three groups: (A) the control group, (B) the humantenirine group and (C) the NMDA group. The mice in group A were intraperitoneally injected with a certain volume of normal saline and then sacrificed. The brain tissue was removed on ice. The mice in group B received an intraperitoneal injection of humantenirine (0.11 mg·kg^−1^). The brain tissues of poisoned dead mice were collected. NMDA (25 mg·kg^−1^) was initially administered to the mice in group C, and humantenirine was given after 20 min. After the mice recovered, they were decapitated, and brain tissue was collected. The hippocampal tissue was separated on ice from the brain tissue of three randomly selected mice in each group for protein expression determination, while the hippocampal tissue of the other mice was prepared for the detection of ATP content and mitochondrial membrane potential.

##### Measurement of ATP Content in the Hippocampal Tissue of Mice

The hippocampal tissue samples were adequately homogenized with cold ATP extract solution after weighing. The homogenate was centrifuged at 8000× *g*/min at 4 °C for 10 min. Then, 0.5 mL chloroform was added to the supernatant, and the well-mixed solution was centrifuged at 10,000× *g* at 4 °C for 3 min. The supernatant was collected and used for the detection of ATP content according to the ATP Assay Kit.

##### Determination of Mitochondrial Membrane Potential

The hippocampal tissues were weighed and homogenized with precooled lysis buffer (1:10, w/v). After centrifugation at 1000× *g*/min at 4 °C for 5 min, the supernatant was collected and centrifuged again at 1000× *g*/min at 4 °C for 5 min. Then, the supernatant was transferred to another 2 mL microfuge tube and centrifuged at 12,000× *g*/min for 10 min. Next, 0.5 mL wash buffer was added to mitochondrial pellets to resuspend, and then centrifuged at 4 °C and 1000× *g* for 5 min. Finally, the supernatant was centrifuged at 12,000× *g*/min for 10 min. The obtained mitochondrial pellets were suspended in store buffer.

The mitochondrial membrane potential was determined by using a JC-1 fluorescent probe. The mitochondrial suspension (20 μL) was added to 180 μL of JC-1 staining working solution (diluted 5 times with JC-1 staining buffer solution). The fluorescence intensity was detected by a fluorescence microplate reader. The excitation wavelength and emission wavelength of J-aggregates (red) were set to 525 nm and 590 nm, respectively. The excitation wavelength and emission wavelength of the JC-1 monomer (green) were set to 490 nm and 530 nm, respectively. The relative ratio of red/green fluorescence intensity was calculated to measure the proportion of mitochondrial depolarization.

##### Detection of the Expression of Key Protein in Mice Hippocampus by Western Blotting

The collected hippocampus samples were weighed and homogenized with RIPA lysis Buffer (Solarbio, China) containing phosphatase inhibitor (Coolaber, China) at low temperature, then lysed in an ice bath for 30 min. Next, the lysate was centrifuged at 13000 rpm at 4 °C for 10 min, and the supernatant was kept. The protein concentration was measured by BCA protein assay kit (CWBIO, China), and the protein samples were stored at −80 °C until use. The protein samples were separated by SDS-PAGE and transferred to PVDF membranes. Then, the membrane was blocked with protein-free rapid blocking buffer on a shaker for 10 min and washed with TBST 3 times, each time for 8 min. The membrane was incubated with the primary antibody (p44/42 MAPK (Erk1/2) (137F5) Rabbit mAb, 1:1000, Cell Signaling; Phospho-p44/42 MAPK (Erk 1/2) (Thr202/Tyr204) Rabbit mAb, 1:1000, Cell Signaling; β-Tubulin rabbit pAb, 1:4000, Proteintech) overnight at 4 °C. After washing with TBST, the appropriate secondary antibody (HRP-labeled goat anti-rabbit IgG (H+L), 1:5000, Biodragon) was added to incubate at room temperature for 1 h. After washing with TBST again, the BLT GelView 6000 Pro imaging system was used to visualize the protein bands.

## 3. Results

### 3.1. Putative Targets of Gelsemium Alkaloids Associated with Excitotoxicity

**Figure 1:**
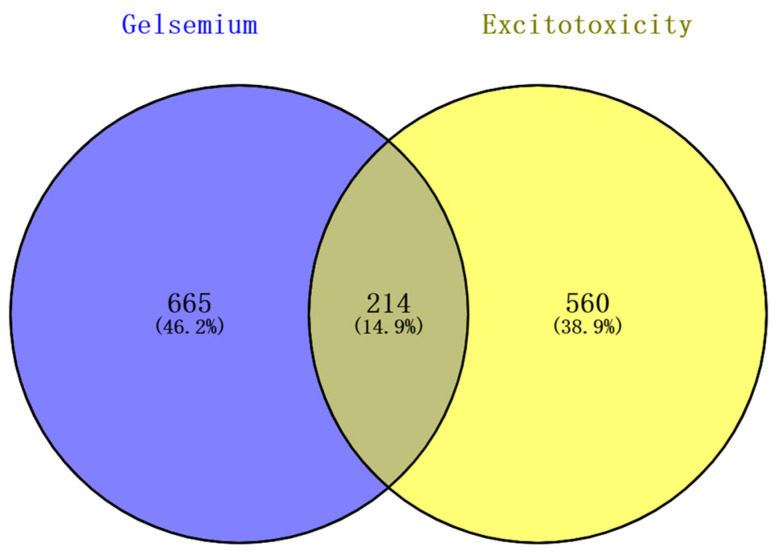
The Venn diagram of excitotoxicity-related targets corresponding to the *Gelsemium* alkaloids.

The 98 alkaloid components that constitute *Gelsemium* were determined by references and databases, and 94 candidate alkaloids were obtained after screening by SwissADME, as shown in Table 1. A total of 879 potential targets corresponding to the alkaloids were predicted based on SwissTargetPrediction and BATMAN-TCM after deleting duplicates (Table S2). A total of 772 and 32 targets related to excitotoxicity were obtained through the GeneCards database and the NCBI database, respectively. After the repetitive targets were removed, 774 targets related to excitotoxicity were finally obtained from the two databases (Table S3). Targets corresponding to alkaloids and excitotoxicity-related targets were intersected using a Venn diagram. The results show that there were 214 intersections of target genes, and these intersections were considered potential candidate targets of excitotoxicity caused by *Gelsemium* (Figure 1).

### 3.2. Construction of a PPI Network of Alkaloid-Excitotoxicity Intersection Targets

**Figure 2:**
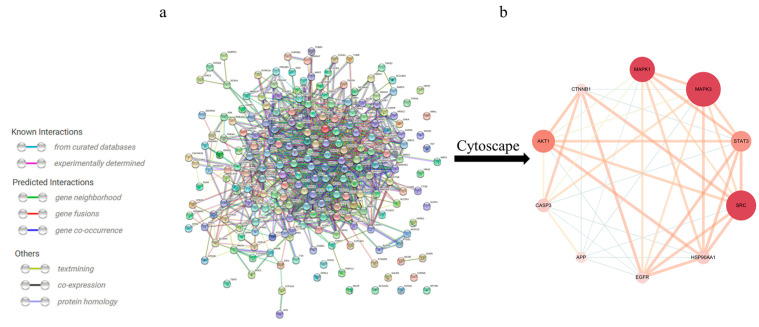
The PPI network for *Gelsemium* alkaloids in excitotoxicity. (**a**) PPI among the 214 core targets. The network nodes represent targets, while the edges represent protein–protein associations. (**b**) PPI network of the core hub targets using Cytoscape 3.6.0 software. The different colors indicate the different importance of the nodes in the whole PPI network. The redder the ellipse is, the more important the node is in the PPI network.

To further identify the core regulatory targets of *Gelsemium* alkaloid-induced excitotoxicity, the STRING database was used to establish a PPI network. With a confidence score > 0.7, the PPI network was composed of 214 nodes and 1233 edges (Figure 2a). The nodes represent proteins, while the edges represent protein–protein interactions. The hub targets with higher Degree, ClosenessCentrality and BetweennessCentrality values were speculated to be the key targets (Table 2), namely, MAPK3, SRC and MAPK1 (Figure 2b).

### 3.3. GO and KEGG Pathway Enrichment Analysis

**Figure 3:**
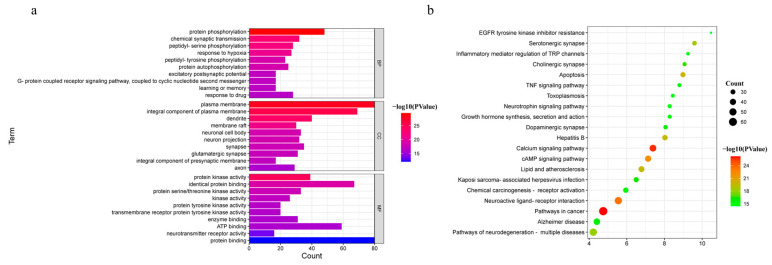
The GO and KEGG pathway enrichment analyses. (**a**) GO analysis of 214 common targets in BP, CC and MF. (**b**) The KEGG pathways based on P-values.

The 214 common targets were input into DAVID (https://david.ncifcrf.gov/, accessed on 02 August 2022) for GO and KEGG pathway enrichment analyses to explore the possible toxic mechanism of *Gelsemium*. Based on the P-value < 0.01, 454 BP, 70 CC and 105 MF terms were enriched in GO analysis (Table S4). The 10 GO terms with the most significant P-values were selected in BP, CC and MF, as shown in Figure 3a. It was suggested that alkaloid-induced excitotoxicity may occur through the regulation of the response to hypoxia, excitatory postsynaptic potential, protein phosphorylation, and others. Meanwhile, the target protein genes were mainly associated with the plasma membrane and participated in protein binding, neurotransmitter receptor activity, ATP binding and protein kinase activity. To further reveal the potential toxic mechanism of *Gelsemium*, KEGG pathway analyses were performed on the 214 targets. A total of 164 pathways were enriched with a P-value < 0.01 (Table S5). The top 20 pathways that were significantly enriched are presented in Figure 3b. It was shown that the toxicity of *Gelsemium* was closely related to pathways that include the calcium signaling pathway, MAPK, cAMP, neuroactive ligand-receptor interaction, apoptosis, long-term potentiation, HIF-1 signaling pathway and serotonergic synapse as well as other synaptic transmission pathways.

### 3.4. Alkaloid–Target and Target–Pathway Network Analysis

**Figure 4:**
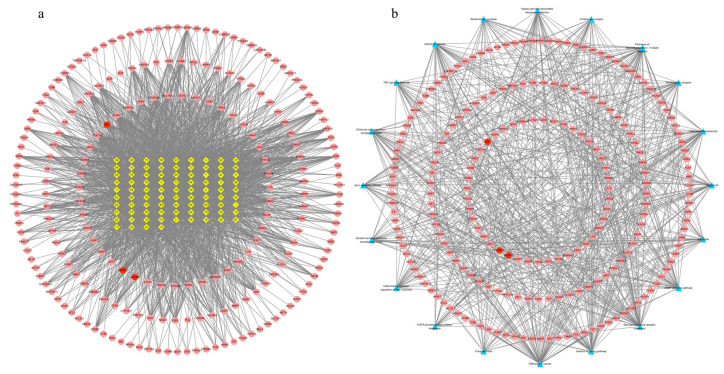
The network for *Gelsemium* alkaloids in excitotoxicity. (**a**) The alkaloid–target network. (**b**) The target–pathway network. The yellow diamonds in the middle indicate *Gelsemium* alkaloids, the red ellipses indicate potential targets of excitotoxicity, and the blue triangles indicate related pathways. See Table 1 for the names of the corresponding alkaloid components.

There were 299 nodes and 2342 edges in the alkaloid–target network (Figure 4a). The nodes included 85 alkaloid component nodes and 214 target nodes, and 20 pathways. The first three alkaloid component nodes with many more edges, which were possibly considered to be the main poisonous components in *Gelsemium*, were 19α-hydroxygelsamydine, gelseiridone and 14-dehydroxygelsefuranidine. The average values of the three topological characteristic parameters (Degree, ClosenessCentrality and BetweennessCentrality) of the three toxic components were 47.3, 0.4109 and 0.0194 respectively. There were 234 nodes and 683 edges in the target–pathway network (Figure 4b). The nodes included 214 target nodes and 20 pathways.

### 3.5. Molecular Docking

**Figure 5:**
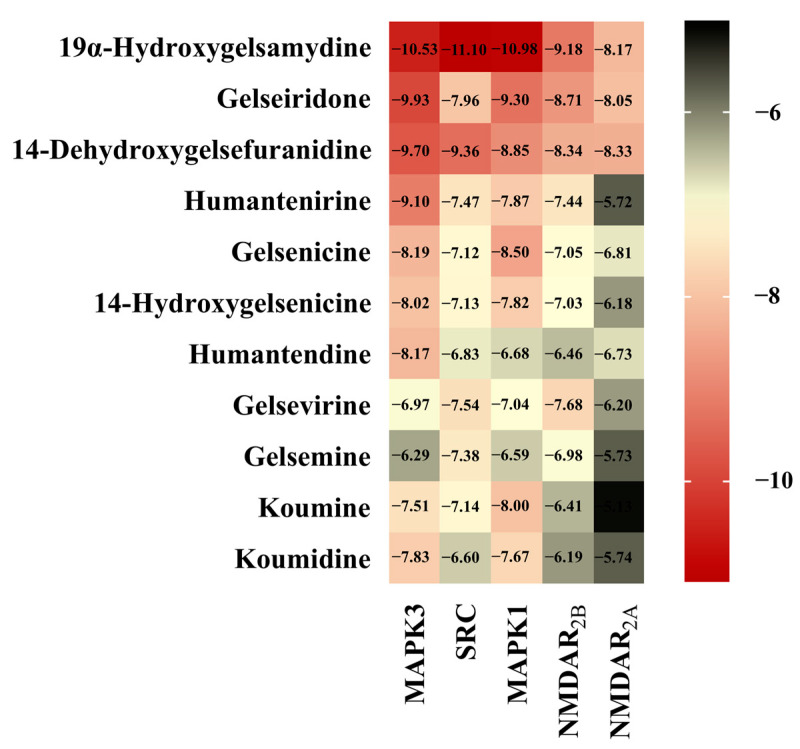
The heatmap of binding energy between *Gelsemium* alkaloids and key targets.

The interaction between alkaloids and core targets was further analyzed by molecular docking. The binding affinity of the ligand and the receptor was evaluated by calculating the binding free energy. The lower the binding energy is, the tighter the binding between the ligand and receptor, the more stable the interaction and the greater the affinity. In addition, the three core targets (MAPK3, SRC and MAPK1) were used as receptors for molecular docking. NMDAR_2B_ and NMDAR_2A_ were also used as receptors since NMDAR is involved in the toxicity of *Gelsemium*, and related genes (GRIN1, GRIN2B and GRIN2A) have also been found in network pharmacology. The first three poisonous ingredients acted as ligands for molecular docking analysis. In addition, humantenirine, gelsenicine, 14-hydroxygelsenicine, humantendine, gelsevirine, gelsemine, koumine and koumidine were also selected as ligands because they are representative and well-studied components of *Gelsemium*. The results show that the binding energy of molecular docking between the alkaloids and the targets was negative, which indicates that all components can spontaneously bind to the targets. The binding energy was less than −5 kcal/mol, indicating that the binding property was good (Figure 5). Next, binding conformations between the alkaloids and the targets were displayed by Discovery Studio 2019.

**Figure 6:**
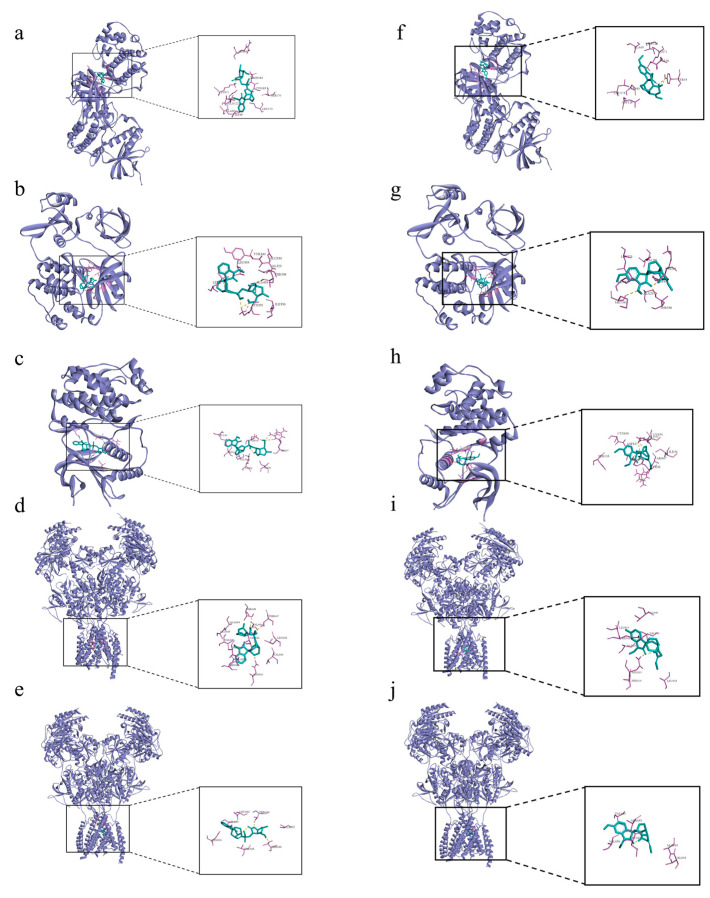
Molecular docking of the binding mode of MAPK3, SRC, MAPK1, NMDA_2B_ and NMDA_2A_ with (**a**–**e**) 19α-hydroxygelsamydineand and (**f**–**j**) humantenirine. The yellow dashed lines represent the hydrogen bonds.

The 19α-hydroxygelsamydine-MAPK3 complex was stabilized via three hydrogen bonds with residues ASP-184 and LYS-71 (Figure 6a). 19α-hydroxygelsamydine fixed the binding cavity of SRC through four H-bonds with residues LYS-295 and THR-338 (Figure 6b). The 19α-hydroxygelsamydine-MAPK1 complex interacted with the residues ASP-167 and ARG-67 through three hydrogen bonds (Figure 6c). 19α-hydroxygelsamydine formed hydrogen bonds with three amino acids, ASN-616, THR-647 and THR-648, to bind to NMDAR_2B_ (Figure 6d). Similarly, the 19α-hydroxygelsamydine–NMDAR_2A_ complex was stabilized by forming two hydrogen bonds with residue THR-646 (Figure 6e). The binding mode between humantenirine and the targets MAPK3, SRC, MAPK1, NMDAR_2B_ and NMDAR_2A_ is shown in Figure 6f-j.

### 3.6. The LD_50_ of Acute Humantenirine Poisoning

**Figure 7:**
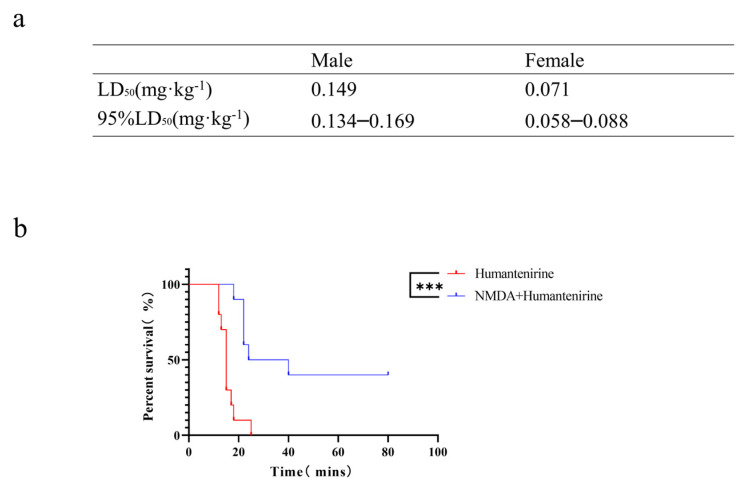
The LD_50_ of humantenirine and the antidotic effect of NMDA on humantenirine poisoning (**a**)The LD_50_ of humantenirine. See also Table S6. (**b**) NMDA significantly increased the survival rate of mice exposed to humantenirine. *** P < 0.01 compared with the humantenirine group.

To verify the accuracy of the network pharmacology analysis, an acute toxicity test of humantenirine was carried out on mice in this study. After intraperitoneal injection of humantenirine, the mice showed symptoms of spontaneous activity reduction, dyspnea, muscle tremor, and then clonic convulsions until death. Humantenirine caused the death of mice in a dose-dependent manner, and most of the deaths occurred within 30 min after administration. In addition, humantenirine showed obvious sex differences. The LD_50_ of female mice was 0.071 mg·kg^−1^, with a 95% confidence interval of 0.058–0.088 mg·kg^−1^, while the LD_50_ of male mice was 0.149 mg·kg^−1^, with a 95 % confidence interval of 0.134–0.169 mg·kg^−1^ (Figure 7a).

### 3.7. The Antidotic Effect of NMDA on Humantenirine Poisoning

An acute poisoning model was used to evaluate the detoxification effects of NMDA on mice poisoned with humantenirine. A survival analysis of the mice was performed, and the survival curve is depicted. There were significant differences in the survival time and survival rate between the control group and the NMDA group. NMDA significantly prolonged the survival time and improved the survival rate of mice (Figure 7b), which indicates that NMDA pre-administration had a protective effect on humantenirine poisoning.

### 3.8. Effect of Humantenirine on ATP

**Figure 8:**
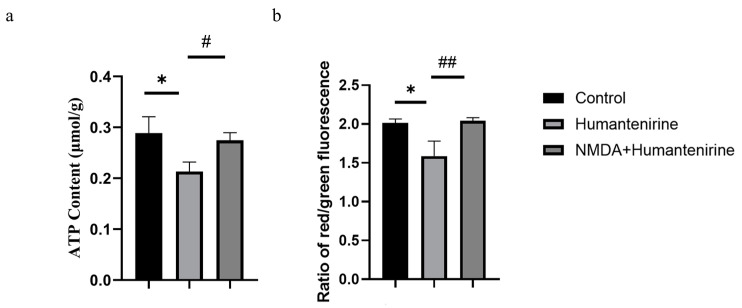
NMDA preventive administration restored mitochondrial function in the hippocampus. (**a**) Decrease of ATP content in the hippocampus after humantenirine administration, while NMDA preventive administration significantly recovered ATP content. (**b**) Decrease of mitochondrial membrane potential in the hippocampus induced by humantenirine after administration, which was significantly improved after NMDA pretreatment. The data are presented as the mean ± SD (n = 3). * P < 0.05 compared with the control group; # P < 0.05 compared with the humantenirine group; ## P < 0.01 compared with the humantenirine group.

ATP is an important energy source for various life activities in the body. To evaluate the effect of humantenirine on ATP content, ATP content in the hippocampus of mice treated with humantenirine was measured and is shown in Figure 8a. Compared with the control group, the content of ATP in the humantenirine group decreased significantly (P < 0.05). However, NMDA preventive administration significantly increased ATP content in the hippocampus (P < 0.05).

### 3.9. Changes in Mitochondrial Membrane Potential

A mitochondrial membrane potential assay kit with JC-1 was used to detect changes in mitochondrial membrane potential. The decrease in the relative ratio of red to green fluorescence indicates a decrease in mitochondrial membrane potential. The results show that the mitochondrial membrane potential in the hippocampus of the humantenirine group decreased significantly (P < 0.05) compared with that in the control group. Compared with the humantenirine group, the level of mitochondrial membrane potential in the hippocampus increased significantly by NMDA preconditioning (P < 0.01) (Figure 8b).

### 3.10. Humantenirine Induced Excitotoxicity by Upregulating Key Target Protein Expression in the Hippocampal Tissue of Mice

**Figure 9:**
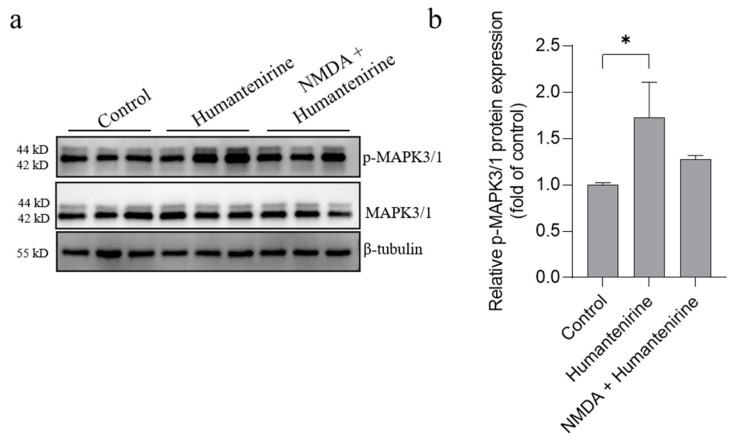
Humantenirine upregulated the phosphorylation level of MAPK3/1. (**a**) Western blot for the expression of proteins including MAPK3/1 and p-MAPK3/1. (**b**) The relative expression of p- MAPK3/1 protein. Data are presented as the mean ± SD (n = 3). * P < 0.05, compared with the control group.

The results of network pharmacology show that MAPK3, SRC and MAPK1 were the key targets of excitotoxicity of *Gelsemium*. Based on our previous studies, we further detected the protein expression levels of MAPK3, MAPK1 and their phosphorylated counterparts by Western blot, and the results are shown in Figure 9. Humantenirine significantly increased the phosphorylation level of MAPK3/1 (P < 0.05). However, there was no significance in protein phosphorylation level between the humantenirine poisoning group and the NDMA pretreatment group.

## 4. Discussion

In this study, a series of bioinformatics methods were combined with experimental verification to systematically study the toxicity mechanism of *Gelsemium* for the first time. A total of 214 potential targets associated with excitotoxicity were distinguished to explore the possible toxic mechanism of *Gelsemium* based on network pharmacological analysis. The first three hub targets, MAPK3 (ERK1), SRC and MAPK1 (ERK2), were regarded as the key targets for the toxic effects of alkaloids in *Gelsemium*. MAPK3 and MAPK1 are the core members of the MAPK family, which play crucial roles in the signal transduction cascade by regulating various cellular processes. The activation of ERK 1/2 can regulate the activity of NMDA receptors by affecting the release of glutamate from synaptosomes, which leads to epilepsy [28,29,30,31]. NMDAR is involved in excitotoxicity and neuronal death under many pathological conditions [32,33]. Treatment with a specific ERK inhibitor can dramatically reduce neuronal cell death induced by excitotoxicity both in vitro and in vivo [34]. SRC belongs to the SRC family of nonreceptor protein tyrosine kinases, is expressed in the central nervous system, and participates in many cellular functions. In the developed central nervous system, SRC can regulate the activities of NMDAR [35], Ca^2+^ voltage-gated ion channels [36] and GABA_A_R [37,38], which have been proven to be closely related to the toxicity of *Gelsemium* [10,13,39]. A previous study showed that lithium had neuroprotective effects against excitotoxicity by regulating the levels of phosphorylated SRC in vitro [40]. In conclusion, it is suggested that *Gelsemium* alkaloids may cause toxic reactions by regulating the activity or phosphorylation of these key targets. This has laid a foundation for further study of the toxic mechanism of *Gelsemium* and the discovery of specific antidotes in the future.

To better understand the complex toxic mechanism of *Gelsemium* from a systematic point of view, GO and KEGG enrichment analyses of the 214 alkaloids corresponding to the excitotoxicity targets were performed. Based on GO functional analysis, it is speculated that *Gelsemium* may cause toxicity by affecting the protein phosphorylation reaction, ATP binding process and plasma membrane function. Protein phosphorylation is a transient post-translational modification that plays an important role in cellular regulation. Most of the aforementioned targets are phosphorylated during activation. For instance, phosphorylated SRC kinase at Tyr416 mediates excitotoxicity [40]. Interestingly, *Gelsemium* affects the phosphorylation of kinases and glutamate receptors, which leads to excitotoxicity [13]. The excitotoxicity elicited by direct exposure to glutamate or other excitotoxic compounds leads to prolonged calcium influx and depolarization of both the cell membrane and mitochondrial membrane [41,42], which is compatible with the results of KEGG enrichment analysis. Additionally, the KEGG pathway enrichment analysis highly enriched the Ca^2+^ signaling pathway and MAPK signaling pathway. Ca^2+^ is a ubiquitous intracellular signal that can regulate cell proliferation, survival, apoptosis and other biological processes. NMDAR possesses a high permeability to sodium and calcium [43]. The continuous increase in glutamate content leads to the overactivation of NMDARs, which promotes the continuous influx of Ca^2+^ into neurons [42]. Subsequently, Ca^2+^ can activate catabolic enzymes that directly cause cell death and tissue damage [44]. In addition, persistent Ca^2+^ influx depletes ATP stores and impairs mitochondrial function, leading to mitochondrial dysfunction and contributing to apoptotic cell death. In addition, previous studies have proven that removal of extracellular calcium could decrease neuronal degeneration induced by excitotoxicity [45]. Ca^2+^ will also affect the release of neurotransmitters [46], which is consistent with our hypothesis that the toxicity of *Gelsemium* derived from the imbalance between neurotransmitters [47]. As for the MAPK signaling pathway, this is another important pathway enriched by KEGG. p38 MAPK signaling plays a key role in NMDAR-mediated apoptosis in striatal neurons [48]. Furthermore, it has been reported that *Gelsemium* can regulate the MAPK signaling pathway in piglets [49]. These findings improve our understanding of the toxic mechanism of *Gelsemium*.

Based on the “alkaloid–target–pathway” network, 85 *Gelsemium* alkaloids associated with excitotoxicity were obtained, including 19α-hydroxygelsamydine, gelseiridone, 14-dehydroxygelsefuranidine, gelselenidine, humantenirine and gelsemolenine B. The combination of these components may lead to the toxicity of *Gelsemium*. The molecular docking results show that 11 alkaloids could autonomously combine with the active pocket of MAPK3, SRC, MAPK1, NMDAR_2B_ and NMDAR_2A_ through hydrogen bonds and form a relatively stable complex. Among these, 19α-hydroxygelsamydine has the lowest binding energies and the highest affinity to these key targets, which indicates that 19α-hydroxygelsamydine was the most toxic potential alkaloid in *Gelsemium*. At the same time, we found that the binding energies of alkaloids with low LD_50_ values, such as gelsenicine and 14-hydroxygelsenicine, to key targets were generally lower than those of alkaloids with high LD_50_ values, including gelsemine, koumine and koumidine (the LD_50_ values of *Gelsemium* alkaloids are shown in Table S5). These findings partly reflect the way in which the difference of binding affinity may be the basis of toxicity differences of *Gelsemium* alkaloids. By analyzing the combination modes of different complexes, it was found that the existence of methoxy and carbonyl groups on the indole nucleus of the more toxic alkaloids, such as humantendine, gelsenicine, 14-hydroxygelsenicine and humantenirine, increased the possibility of hydrogen bonding with the target proteins, which increased the affinity between the ligand and receptor. This result is closely related to a previous report that the methoxy group at N1 is the structural basis of *Gelsemium* toxicity [50]. Furthermore, the binding energy of alkaloids with NMDAR_2B_ was generally lower than that with NMDAR_2A_, indicating that *Gelsemium* alkaloids had a stronger affinity for NMDAR_2B_. This is consistent with previous research, which showed that the stimulation of NMDAR containing the N2B subunit will lead to the activation of the excitotoxic pathway that leads to neuron death instead of NMDAR containing the N2A subunit [51]. Taken together, these findings indicate that *Gelsemium* may regulate the function of NMDAR by acting on MAPK3, SRC and MAPK1 targets or by directly binding to NMDAR.

Although 19α-hydroxygelsamydine showed specific toxicity potential in network pharmacology, it is not the most abundant of the alkaloids in *Gelsemium*. As it is difficult to obtain, there is no report on its toxicity. At present, it has been found that the extracts and monomers derived from *Gelsemium* possess potential toxicity, and the toxicity of gelsenicine (0.185 mg·kg^−1^ i.p.) [6,39], 14-hydroxygelsenicine (0.295 mg·kg^−1^ p.o.) [10], humantendine (0.21 mg·kg^−1^ i.p.) [7] and gelsemine (56.2 mg·kg^−1^ i.p.) [5] have been well characterized, while the toxicity of humantenirine is still ambiguous. Therefore, humantenirine was chosen as a substitute, based on its relative accessibility, to evaluate both the toxicity and the toxic mechanism in the experimental verification. In the current study, the acute toxicity of humantenirine was investigated in mice to further verify the results of network pharmacology. After intraperitoneal injection of humantenirine, mice developed symptoms of respiratory depression and convulsion and died within 30 min. Furthermore, humantenirine showed significant sex differences, and the LD_50_ values of female and male mice were 0.071 mg·kg^−1^ and 0.149 mg·kg^−1^, respectively. By analyzing the LD_50_ of different alkaloids, we found that humantenirine is a highly toxic alkaloid, and its toxicity is equivalent to that of gelsenicine, an alkaloid of gelsedine-type with strong toxicity. In our previous study, we found that excitotoxicity was crucial to the toxicity of gelsenicine and that NMDA (a specific NMDAR agonist) could protect against gelsenicine poisoning [13]. In addition, evidence has shown that NMDA can protect neurons against excitotoxicity by regulating extracellular glutamate concentrations and maintaining intracellular Ca^2+^ homeostasis [52,53]. Therefore, the detoxification of NMDA in humantenirine poisoning was evaluated. The survival curve showed that NMDA pretreatment could significantly improve the survival rate of humantenirine-poisoned mice. This may further indicate that the toxicity of *Gelsemium* was caused by excitotoxicity. Some studies have shown that excitotoxicity could lead to mitochondrial damage [54,55], which is consistent with experimental results that show that humantenirine could significantly decrease ATP content and mitochondrial membrane potential in hippocampal tissue, the key brain region of *Gelsemium* toxicity, in mice. Recently, it has been shown that the level of phosphorylated ERK could be increased in glutamate-induced excitotoxicity [56]. Furthermore, protection with MAPK/ERK kinase specific inhibitor in both HT22 cells and immature primary cultured cortical neurons could inhibit ERK1/2 phosphorylation against oxidative stress, which is implicated in the pathogenesis of neuronal degeneration [57] as well as in the specific inhibitor against oxidative glutamate toxicity in HT22 cells [58]. Our experimental results suggest that humantenirine upregulates the phosphorylation level of MAPK3/1, causing excitotoxicity and then mitochondrial dysfunction. As for the detoxification of NMDA, we speculate that this was related to the recovery of mitochondrial function in some way, instead of inhibiting the phosphorylation of MAPK3/1. Although SRC has not been studied here, we cannot rule out the idea that it plays an important role in the toxicity of *Gelsemium.* Based on the similarity of clinical poisoning symptoms and skeletal structure, it is reasonable to speculate that humantenirine, or other alkaloid monomers derived from *Gelsemium*, may act on some excitotoxicity-related targets, leading to overactivation of NMDARs, which in turn leads to mitochondrial dysfunction of hippocampus and death of mice, but further in-depth research on its mechanism is needed.

## 5. Conclusions

This study integrated a series of bioinformatic methods with experimental verification to systematically explore the toxic mechanism of *Gelsemium*. We have demonstrated that *Gelsemium* alkaloids may induce excitotoxicity by regulating the phosphorylation levels of key targets such as MAPK3 and MAPK1, thus causing mitochondrial dysfunction and leading to death. However, NMDA rescued humantenirine-induced excitotoxicity by restoring the function of mitochondria rather than inhibiting the phosphorylation of MAPK3/1. The present study is helpful in expanding the understanding of the toxic mechanism of *Gelsemium* and lays the foundation for its development and utilization. Our research further shows that the combination of network pharmacological analysis and experimental verification may provide a favorable means to elucidate the mechanism of action of drugs.

## Figures and Tables

**Table 1:** The information of the alkaloids in *Gelsemium* (the structures of the corresponding alkaloids were shown in Table S1).

NO.	Compound	CID	MW	MF
1	11-Hydroxyhumantenine	5318224	370.4	C_21_H_26_N_2_O_4_
2	11-Hydroxyrankinidine	5318332	356.4	C_20_H_24_N_2_O_4_
3	11-Methoxy-19-(R)-Hydroxygelselegine	5319453	404.5	C_21_H_28_N_2_O_6_
4	11-Methoxygelsemamide	5319437	355.4	C_21_H_25_NO_4_
5	11-Methoxyhumantenine	44583832	384.5	C_22_H_28_N_2_O_4_
6	14β-Hydroxygelsedine	126023	344.4	C_19_H_24_N_2_O_4_
7	15-Hydroxyhumantenine	101606434	370.4	C_21_H_26_N_2_O_4_
8	16-Epi-Voacarpine	5317127	368.4	C_21_H_24_N_2_O_4_
9	19-(R)-Hydroxydihydrogelsemine	5318191	340.4	C_20_H_24_N_2_O_3_
10	19-(R)-Hydroxydihydrogelsevirine	5318192	370.4	C_21_H_26_N_2_O_4_
11	19-(R)-Hydroxydihydrokoumine	5318193	324.4	C_20_H_24_N_2_O_2_
12	19-(S)-Hydroxydihydrogelsevirine	5318192	370.4	C_21_H_26_N_2_O_4_
13	19-(S)-Hydroxydihydrokoumine	5318193	324.4	C_20_H_24_N_2_O_2_
14	19-(Z)-Akuammidine	44583830	352.4	C_21_H_24_N_2_O_3_
15	19-(Z)-Taberpsychine	5321582	310.4	C_20_H_26_N_2_O
16	19-Oxo-Gelsenicine	5320330	340.4	C_19_H_20_N_2_O_4_
17	20-Hydroxydihydrorankinidine	101606432	358.4	C_20_H_26_N_2_O_4_
18	Akuammidine N-Oxide	11268654	368.4	C_21_H_24_N_2_O_4_
19	Akuammidinen-Oxide	102423744	368.4	C_21_H_24_N_2_O_4_
20	Dihydrokoumine	5316727	308.4	C_20_H_24_N_2_O
21	Elegansamine	5317023	508.6	C_29_H_36_N_2_O_6_
22	Epiwilsonine	5315317	343.4	C_20_H_25_NO_4_
23	Gelsamydine	5317540	508.6	C_29_H_36_N_2_O_6_
24	Gelsedine	21589070	328.4	C_19_H_24_N_2_O_3_
25	Gelsemamide	5317542	340.4	C_20_H_24_N_2_O_3_
26	Gelsemicine	5462428	358.4	C_20_H_26_N_2_O_4_
27	Gelsemine	5390854	322.4	C_20_H_22_N_2_O_2_
28	4-(S)-Gelsemine N-Oxide	5317545	338.4	C_20_H_22_N_2_O_3_
29	4-(R)-Gelsemine N-Oxide	5317545	338.4	C_20_H_22_N_2_O_3_
30	Gelsemoxonine	44583831	358.4	C_19_H_22_N_2_O_5_
31	Gelsevirine	14217344	352.4	C_21_H_24_N_2_O_3_
32	Humantenidine	44584549	342.4	C_19_H_22_N_2_O_4_
33	Humantenine	44593672	354.4	C_21_H_26_N_2_O_3_
34	Humantenirine	11132403	370.4	C_21_H_26_N_2_O_4_
35	Humantenmine	158212	326.4	C_19_H_22_N_2_O_3_
36	Koumidine	44584550	294.4	C_19_H_22_N_2_O
37	Koumine N-Oxide	5318847	322.4	C_20_H_22_N_2_O_2_
38	N-Desmethoxyhumantenine	5316593	324.4	C_20_H_24_N_2_O_2_
39	N-Desmethoxyrankinidine	5316594	310.4	C_19_H_22_N_2_O_2_
40	Oxoglaucine	97662	351.4	C_20_H_17_NO_5_
41	Rankinidine	6439112	340.4	C_20_H_24_N_2_O_3_
42	Sempervirine(ii)	168919	272.3	C_19_H_16_N_2_
43	Tabersonine	25201472	337.4	C_21_H_25_N_2_O_2_^+^
44	14-Hydroxygelsemicine	597741	374.4	C_20_H_26_N_2_O_5_
45	Gelsenicine	21123652	326.4	C_19_H_22_N_2_O_3_
46	Gelegamine D	101467880	356.4	C_20_H_24_N2O_4_
47	Gelegamine E	101467881	370.4	C_20_H_22_N_2_O_5_
48	GS-1	12070887	386.4	C_20_H_22_N_2_O_6_
49	GS-2	12070888	372.4	C_20_H_24_N_2_O_5_
50	11-Hydroxygelsenicine	102004554	342.4	C_19_H_22_N_2_O_4_
51	11,14-Dihydroxygelsenicine	101727430	358.4	C_19_H_22_N_2_O_5_
52	14-Hydroxygelsenicine	14217347	342.4	C_19_H_22_N_2_O_4_
53	14-Acetoxygelsenicine	11962104	384.4	C_21_H_24_N_2_O_5_
54	14,15-Dihydroxygelsenicine	44583829	358.4	C_19_H_22_N_2_O_5_
55	Gelsedilam	102254466	314.34	C_17_H_18_N_2_O_4_
56	Gelsecrotonidine	101449927	396.4	C_22_H_24_N_2_O_5_
57	14-Hydroxygelsecrotonidine	101449929	412.4	C_22_H_24_N_2_O_6_
58	11-Methoxygelsecrotonidine	101449930	426.5	C_23_H_26_N_2_O_6_
59	14α-Hydroxygelsamydine	44559138	524.6	C_29_H_36_N_2_O_7_
60	19α-Hydroxygelsamydine	102003053	524.6	C_29_H_36_N_2_O_7_
61	Gelegamine C	101467879	514.4	C_21_H_27_IN_2_O_5_
62	14-Acetoxygelselegine	101727431	430.5	C_23_H_30_N_2_O_6_
63	14α-Hydroxyelegansamine	44559137	524.6	C_29_H_36_N_2_O_7_
64	Gelseoxazolidinine	102297300	428.5	C_23_H_28_N_2_O_6_
65	Gelseziridine	101951238	342.4	C_19_H_22_N_2_O_4_
66	GS-3	101751032	388.4	C_20_H_24_N_2_O_6_
67	Gelselenidine	101951237	368.4	C_21_H_24_N_2_O_4_
68	Gelsesyringalidine	136704418	490.5	C_28_H_30_N_2_O_6_
69	Gelsevanillidine	136811988	460.5	C_27_H_28_N_2_O_5_
70	Gelsefuranidine	102254468	420.5	C_24_H_24_N_2_O_5_
71	14-Dehydroxygelsefuranidine	102417029	404.5	C_24_H_24_N_2_O_4_
72	Gelsemolenine A	101951239	384.4	C_21_H_24_N_2_O_5_
73	Gelsemolenine B	101951240	370.4	C_20_H_22_N_2_O_5_
74	Gelseiridone	101397829	538.6	C_29_H_34_N_2_O_8_
75	21-Oxogelsemine	11078214	336.4	C_20_H_20_N_2_O_3_
76	21-Oxogelsevirine	184299	366.4	C_21_H_22_N_2_O_4_
77	Gelsebanine	16086585	504.6	C_30_H_36_N_2_O_5_
78	6-Hydroxyhumantenine	101855842	370.4	C_21_H_26_N_2_O_5_
79	19(E)-Humantenine	101520842	354.4	C_21_H_26_N_2_O_3_
80	Gelegamine A	101467877	384.4	C_21_H_24_N_2_O_5_
81	Gelegamine B	101467878	384.4	C_21_H_24_N_2_O_6_
82	Kounaminal	102260292	363.5	C_22_H_25_N_3_O_2_
83	Gelsempervine A	131636659	382.5	C_22_H_26_N_2_O_4_
84	Gelsempervine B	101727385	424.5	C_24_H_28_N_2_O_5_
85	Gelsempervine C	12444814	382.5	C_22_H_26_N_2_O_4_
86	Gelsempervine D	101744809	424.5	C_24_H_28_N_2_O_5_
87	N-Methoxyanhydrovobasinediol	102004539	338.4	C_21_H_26_N_2_O_2_
88	Dehydrokoumidine	119077162	292.4	C_19_H_20_N_2_O
89	Sempervilam	11483103	288.3	C_19_H_16_N_2_O
90	Ourouparine	71436261	329.4	C_21_H_17_N_2_O_2_^+^
91	Gelsebamine	16086588	255.35	C_14_H_25_NO_3_
92	Koumine	91895267	306.4	C_20_H_22_N_2_O
93	Humantendine	5490912	342.4	C_19_H_22_N_2_O_4_
94	Gelsevirine N-Oxide	101951241	368.4	C_21_H_24_N_2_O_4_

**Table 2:** The key targets of the PPI network.

NO.	Name	BetweennessCentrality	ClosenessCentrality	Degree
1	MAPK3	0.0634	0.5272	60
2	SRC	0.0695	0.5105	56
3	MAPK1	0.0381	0.5013	52

## Data Availability

Not applicable.

## Supplementary Materials

The following supporting information can be downloaded at: https://www.mdpi.com/article/10.3390/metabo13020195/s1, Table S1: The information of the alkaloids in *Gelsemium*; Table S2: The predicted targets of *Gelsemium* alkaloids; Table S3: The targets related to excitotoxicity; Table S4: The GO analysis of 214 intersective targets based on the P-value < 0.01; Table S5: The KEGG pathway enrichment analysis of 214 intersective targets based on the P-value < 0.01; Table S6: Acute toxicity of humantenirine in ICR male and female mice; Table S7: The LD_50_ of *Gelsemium* alkaloids in mice.
