# Supplementary material for: Network Pharmacology and Experimental Verification to Unveil the Mechanism of N-Methyl-D-Aspartic Acid Rescue Humantenirine-Induced Excitotoxicity
Source: Metabolites. 2023 Jan 28;13(2):195. doi: 10.3390/metabo13020195 (PMC9966887; doi:10.3390/metabo13020195)
Supplement: Supplementary file 1 [file metabolites-13-00195-s001.zip › metabolites-2111174-supplementary.pdf]

Network pharmacology and experimental verification to unveil the mechanism of NMDA rescue humantenirine-induced excitotoxicity

Xue-Jia Qi[1,2], Chong-Yin Huang[1,2], Meng-Ting Zuo[1,2], Meng-Die Gong[1,2], Si-Juan Huang[1,2], Mo-Huan Tang[1,2], Zhao-Ying Liu [1,2*]

1 College of Veterinary Medicine, Hunan Agricultural University, Changsha 410128, Hunan, China

2 Hunan Engineering Technology Research Center of Veterinary Drugs, Hunan Agricultural University, Changsha 410128, Hunan, China

*** Corresponding author:** Dr. Zhao-Ying Liu,

Hunan Agricultural University,

Furong District,

Changsha 410128,

Hunan, China.

E-mail: liu_zhaoying@hunau.edu.cn (Z.Y. Liu)

Table S1: The information of the alkaloids in *Gelsemium*.

NO.	Compound	CID	MW	MF	Structure
1	11-Hydroxyhumantenine	5318224	370.4	$C_{21}H_{26}N_2O_4$	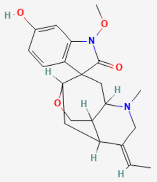
2	11-Hydroxyrankinidine	5318332	356.4	$C_{20}H_{24}N_2O_4$	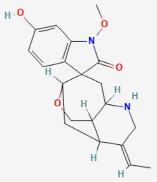
3	11-Methoxy-19-(R)-Hydroxygelselegine	5319453	404.5	$C_{21}H_{28}N_2O_6$	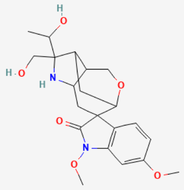
4	11-Methoxygelsemamide	5319437	355.4	$C_{21}H_{25}NO_4$	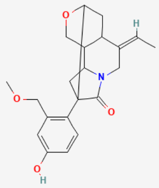
5	11-Methoxyhumantenine	44583832	384.5	$C_{22}H_{28}N_2O_4$	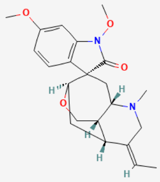
6	14β-Hydroxygelsedine	126023	344.4	$C_{19}H_{24}N_2O_4$	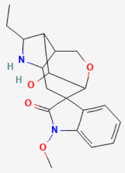
7	15-Hydroxyhumantenine	101606434	370.4	$C_{21}H_{26}N_2O_4$	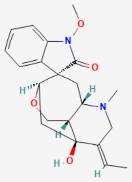
8	16-Epi-Voacarpine	5317127	368.4	$C_{21}H_{24}N_2O_4$	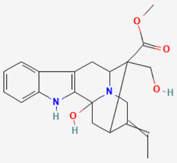

9	19-(R)-Hydroxydihydrogelsemine	5318191	340.4	$C_{20}H_{24}N_2O_3$	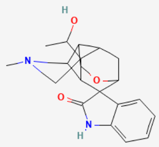
10	19-(R)-Hydroxydihydrogelsevirine	5318192	370.4	$C_{21}H_{26}N_2O_4$	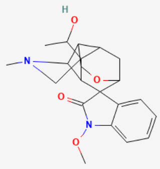
11	19-(R)-Hydroxydihydrokoumine	5318193	324.4	$C_{20}H_{24}N_2O_2$	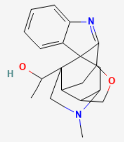
12	19-(S)-Hydroxydihydrogelsevirine	5318192	370.4	$C_{21}H_{26}N_2O_4$	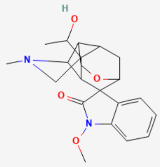
13	19-(S)-Hydroxydihydrokoumine	5318193	324.4	$C_{20}H_{24}N_2O_2$	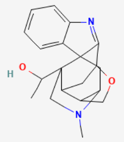
14	19-(Z)-Akuammidine	44583830	352.4	$C_{21}H_{24}N_2O_3$	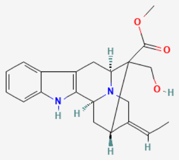
15	19-(Z)-Taberpsychine	5321582	310.4	$C_{20}H_{26}N_2O$	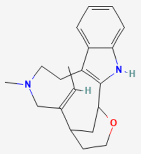
16	19-Oxo-Gelsenicine	5320330	340.4	$C_{19}H_{20}N_2O_4$	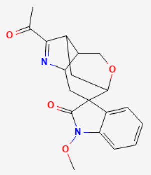

17	20-Hydroxydihydrorankinidine	101606432	358.4	$C_{20}H_{26}N_2O_4$	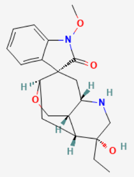
18	Akuammidine N-Oxide	11268654	368.4	$C_{21}H_{24}N_2O_4$	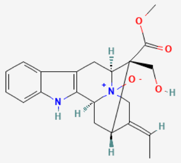
19	Akuammidinen-Oxide	102423744	368.4	$C_{21}H_{24}N_2O_4$	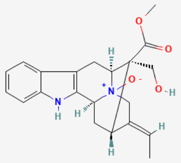
20	Dihydrokoumine	5316727	308.4	$C_{20}H_{24}N_2O$	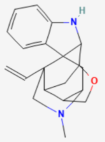
21	Elegansamine	5317023	508.6	$C_{29}H_{36}N_2O_6$	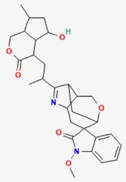
22	Epiwilsonine	5315317	343.4	$C_{20}H_{25}NO_4$	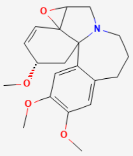
23	Gelsamydine	5317540	508.6	$C_{29}H_{36}N_2O_6$	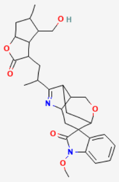
24	Gelsedine	21589070	328.4	$C_{19}H_{24}N_2O_3$	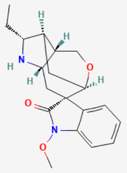

25	Gelsemamide	5317542	340.4	$C_{20}H_{24}N_2O_3$	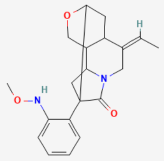
26	Gelsemicine	5462428	358.4	$C_{20}H_{26}N_2O_4$	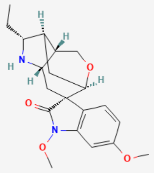
27	Gelsemine	5390854	322.4	$C_{20}H_{22}N_2O_2$	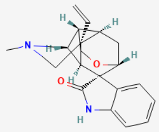
28	4-(S)-Gelsemine N-Oxide	5317545	338.4	$C_{20}H_{22}N_2O_3$	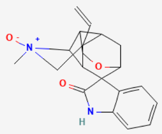
29	4-(R)-Gelsemine N-Oxide	5317545	338.4	$C_{20}H_{22}N_2O_3$	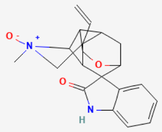
30	Gelsemoxonine	44583831	358.4	$C_{19}H_{22}N_2O_5$	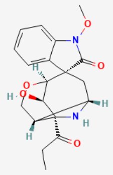
31	Gelsevirine	14217344	352.4	$C_{21}H_{24}N_2O_3$	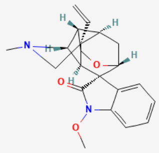
32	Humantenidine	44584549	342.4	$C_{19}H_{22}N_2O_4$	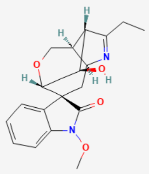

33	Humantenine	44593672	354.4	$C_{21}H_{26}N_2O_3$	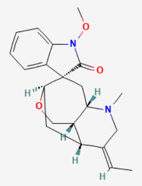
34	Humantenirine	11132403	370.4	$C_{21}H_{26}N_2O_4$	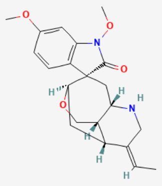
35	Humantenmine	158212	326.4	$C_{19}H_{22}N_2O_3$	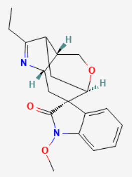
36	Koumidine	44584550	294.4	$C_{19}H_{22}N_2O$	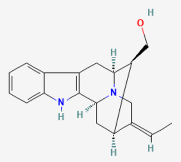
37	Koumine N-Oxide	5318847	322.4	$C_{20}H_{22}N_2O_2$	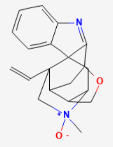
38	N-Desmethoxyhumantenine	5316593	324.4	$C_{20}H_{24}N_2O_2$	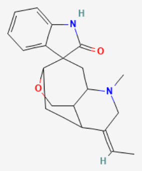
39	N-Desmethoxyrankinidine	5316594	310.4	$C_{19}H_{22}N_2O_2$	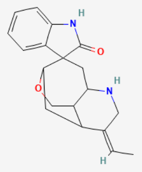
40	Oxoglaucine	97662	351.4	$C_{20}H_{17}NO_5$	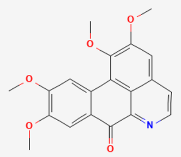

41	Rankinidine	6439112	340.4	$C_{20}H_{24}N_2O_3$	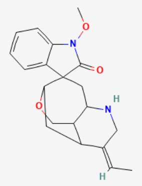
42	Sempervirine(ii)	168919	272.3	$C_{19}H_{16}N_2$	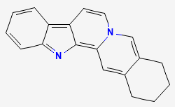
43	Tabersonine	25201472	337.4	$C_{21}H_{25}N_2O_2^+$	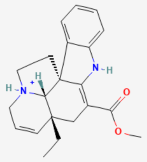
44	14-Hydroxygelsemicine	597741	374.4	$C_{20}H_{26}N_2O_5$	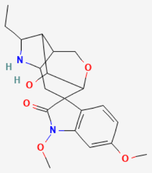
45	Gelsenicine	21123652	326.4	$C_{19}H_{22}N_2O_3$	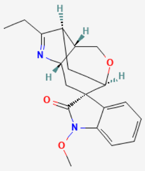
46	Gelegamine D	101467880	356.4	$C_{20}H_{24}N2O_4$	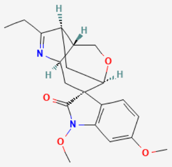
47	Gelegamine E	101467881	370.4	$C_{20}H_{22}N_2O_5$	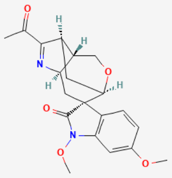
48	GS-1	12070887	386.4	$C_{20}H_{22}N_2O_6$	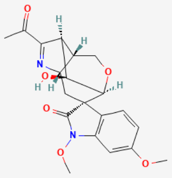

49	GS-2	12070888	372.4	$C_{20}H_{24}N_2O_5$	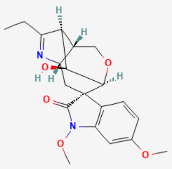
50	11-Hydroxygelsenicine	102004554	342.4	$C_{19}H_{22}N_2O_4$	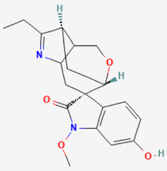
51	11,14-Dihydroxygelsenicine	101727430	358.4	$C_{19}H_{22}N_2O_5$	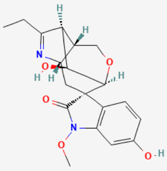
52	14-Hydroxygelsenicine	14217347	342.4	$C_{19}H_{22}N_2O_4$	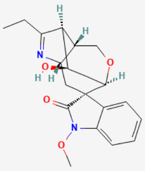
53	14-Acetoxygelsenicine	11962104	384.4	$C_{21}H_{24}N_2O_5$	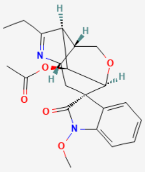
54	14,15-Dihydroxygelsenicine	44583829	358.4	$C_{19}H_{22}N_2O_5$	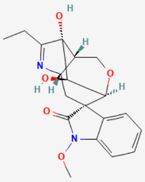
55	Gelsedilam	102254466	314.34	$C_{17}H_{18}N_2O_4$	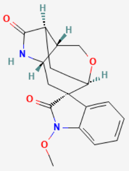
56	Gelsecrotonidine	101449927	396.4	$C_{22}H_{24}N_2O_5$	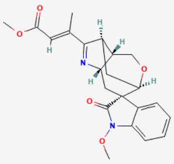

57	14-Hydroxygelsecrotonidine	101449929	412.4	$C_{22}H_{24}N_2O_6$	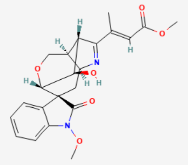
58	11-Methoxygelsecrotonidine	101449930	426.5	$C_{23}H_{26}N_2O_6$	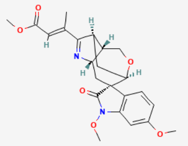
59	14α-Hydroxygelsamydine	44559138	524.6	$C_{29}H_{36}N_2O_7$	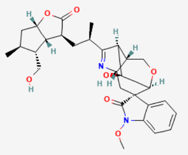
60	19α-Hydroxygelsamydine	102003053	524.6	$C_{29}H_{36}N_2O_7$	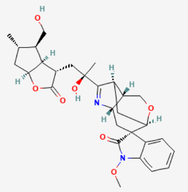
61	Gelegamine C	101467879	514.4	$C_{21}H_{27}IN_2O_5$	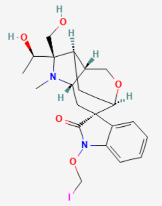
62	14-Acetoxygelselegine	101727431	430.5	$C_{23}H_{30}N_2O_6$	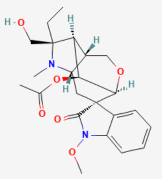
63	14α-Hydroxyelegansamine	44559137	524.6	$C_{29}H_{36}N_2O_7$	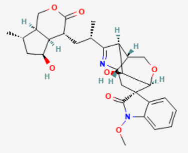
64	Gelseoxazolidinine	102297300	428.5	$C_{23}H_{28}N_2O_6$	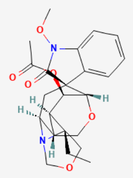

65	Gelseziridine	101951238	342.4	$C_{19}H_{22}N_2O_4$	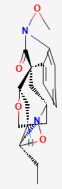
66	GS-3	101751032	388.4	$C_{20}H_{24}N_2O_6$	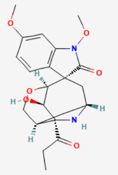
67	Gelselenidine	101951237	368.4	$C_{21}H_{24}N_2O_4$	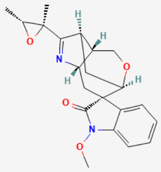
68	Gelsesyringalidine	136704418	490.5	$C_{28}H_{30}N_2O_6$	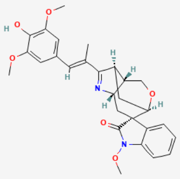
69	Gelsevanillidine	136811988	460.5	$C_{27}H_{28}N_2O_5$	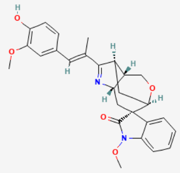
70	Gelsefuranidine	102254468	420.5	$C_{24}H_{24}N_2O_5$	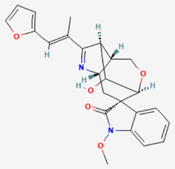
71	14-Dehydroxygelsefuranidine	102417029	404.5	$C_{24}H_{24}N_2O_4$	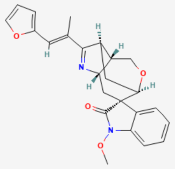
72	Gelsemolenine A	101951239	384.4	$C_{21}H_{24}N_2O_5$	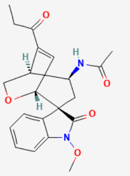

73	Gelsemolenine B	101951240	370.4	$C_{20}H_{22}N_2O_5$	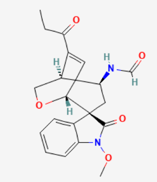
74	Gelseiridone	101397829	538.6	$C_{29}H_{34}N_2O_8$	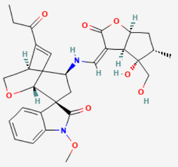
75	21-Oxogelsemine	11078214	336.4	$C_{20}H_{20}N_2O_3$	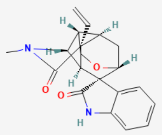
76	21-Oxogelsevirine	184299	366.4	$C_{21}H_{22}N_2O_4$	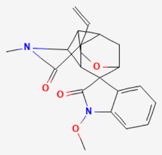
77	Gelsebanine	16086585	504.6	$C_{30}H_{36}N_2O_5$	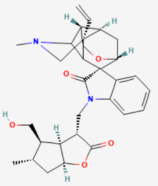
78	6-Hydroxyhumantenine	101855842	370.4	$C_{21}H_{26}N_2O_5$	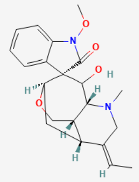
79	19(E)-Humantenine	101520842	354.4	$C_{21}H_{26}N_2O_3$	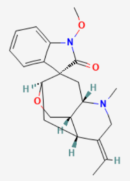
80	Gelegamine A	101467877	384.4	$C_{21}H_{24}N_2O_5$	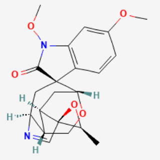

81	Gelegamine B	101467878	384.4	$C_{21}H_{24}N_2O_6$	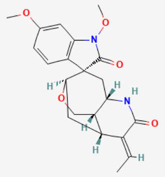
82	Kounaminal	102260292	363.5	$C_{22}H_{25}N_3O_2$	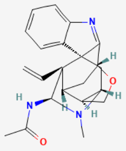
83	Gelsempervine A	131636659	382.5	$C_{22}H_{26}N_2O_4$	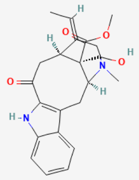
84	Gelsempervine B	101727385	424.5	$C_{24}H_{28}N_2O_5$	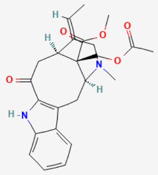
85	Gelsempervine C	12444814	382.5	$C_{22}H_{26}N_2O_4$	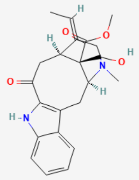
86	Gelsempervine D	101744809	424.5	$C_{24}H_{28}N_2O_5$	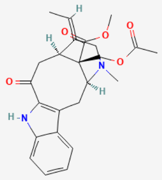
87	N-Methoxyanhydrovobasinediol	102004539	338.4	$C_{21}H_{26}N_2O_2$	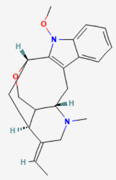
88	Dehydrokoumidine	119077162	292.4	$C_{19}H_{20}N_2O$	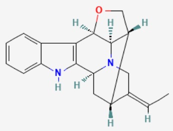

89	Sempervilam	11483103	288.3	$C_{19}H_{16}N_2O$	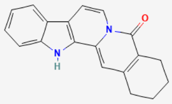
90	Ourouparine	71436261	329.4	$C_{21}H_{17}N_2O_2^+$	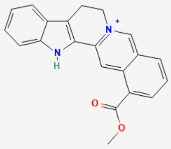
91	Gelsebamine	16086588	255.35	$C_{14}H_{25}NO_3$	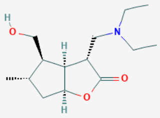
92	Koumine	91895267	306.4	$C_{20}H_{22}N_2O$	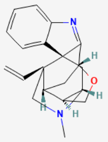
93	Humantendine	5490912	342.4	$C_{19}H_{22}N_2O_4$	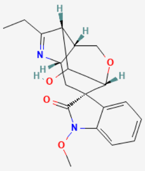
94	Gelsevirine N-Oxide	101951241	368.4	$C_{21}H_{24}N_2O_4$	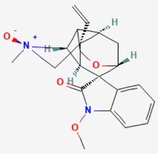

Table S2. The predicted targets of *Gelsemium* alkaloids.

NO.	Targets	NO.	Targets
1	TLR4	441	GCK
2	CHRM3	442	CAPN1
3	DRD1	443	CDK6
4	CHRM1	444	BCL2
5	KCNA5	445	RORC
6	DRD2	446	CAPN2
7	LAP3	447	ABL1
8	PEPD	448	MST1R
9	ADRA2A	449	FGFR3
10	ADRA2C	450	PLK1
11	ADRA2B	451	PRKCZ
12	ADRA1A	452	TEK
13	SIGMAR1	453	MAP3K5
14	HDAC6	454	WNK2
15	HDAC1	455	NEK1
16	DRD3	456	PLK2
17	CHRM4	457	STK38
18	F2	458	HIPK4
19	F10	459	ERN1
20	TACR1	460	OXSR1
21	MTOR	461	MAK
22	XIAP	462	STK39
23	BIRC2	463	CDKL3
24	CHRNA4	464	PIK3C2G
25	JAK1	465	MAP3K6
26	COMT	466	MAP3K13
27	HTR1B	467	MAP3K15
28	HTR1D	468	MAST1
29	CHRM5	469	SBK1
30	FDFT1	470	HUNK
31	HRH4	471	CDK13
32	ERBB2	472	FLT1
33	EGFR	473	RAF1
34	PIM1	474	CASK
35	PIM2	475	SNRK
36	PRKCD	476	DSTYK
37	PRKCQ	477	FLT4
38	BRD4	478	IKBKB
39	ACHE	479	EPHA2
40	PIK3CA	480	MAP2K3
41	HTR2C	481	PRKAA2
42	ESR1	482	CCNH

43	STAT3	483	CCNT1
44	METAP2	484	CYP11B2
45	OPRM1	485	STK26
46	DPP4	486	CNR2
47	HTR6	487	PFKFB3
48	KDR	488	HSD17B2
49	PIK3CG	489	EPHX1
50	DHFR	490	SCN10A
51	HRH1	491	BRS3
52	CDK9	492	NR1H4
53	CHRNB2	493	RHOA
54	PIK3R1	494	TTK
55	SRC	495	NAMPT
56	PDE10A	496	MCL1
57	ADRB2	497	AKR1C3
58	PDE5A	498	PTK2B
59	PDE11A	499	TSPO
60	BCHE	500	AOC3
61	SCN5A	501	NR4A1
62	ADRB1	502	IDH1
63	ADRB3	503	SLC27A1
64	GRIN2B	504	FNTB
65	IRAK4	505	TRPM8
66	CHEK1	506	NPY1R
67	DRD4	507	CA2
68	HTR1A	508	MAP3K14
69	TRPV3	509	CA7
70	SLC18A3	510	CA3
71	LRRK2	511	CA6
72	SLC6A9	512	CA12
73	XPNPEP1	513	CA9
74	XPNPEP2	514	NQO1
75	PRMT6	515	PARP3
76	HTR4	516	MAPKAPK2
77	NR3C1	517	CDC25A
78	REN	518	NUDT1
79	PRMT8	519	HMOX1
80	PRMT1	520	CASP1
81	HTR2B	521	CES1
82	SYK	522	CES2
83	ZAP70	523	VCAM1
84	JAK2	524	SCN9A
85	LYN	525	IMPDH2
86	PNMT	526	GLRA1

87	ADRA1D	527	GLRA2
88	GSK3B	528	PDE4B
89	CYP2D6	529	PDE7A
90	ADRA1B	530	GABRA2
91	AKT1	531	PTAFR
92	CTSD	532	KCNJ11
93	MAPK1	533	FAAH
94	LTA4H	534	CYP17A1
95	MAP3K12	535	GABRA3
96	OGFRL1	536	SGK1
97	HRH3	537	PHKG2
98	MAPK14	538	CSNK1G1
99	ADORA2A	539	CAMK4
100	PARP1	540	TLR9
101	PTPN1	541	SSTR3
102	AURKB	542	CYP1A2
103	NOS1	543	TGFBR1
104	MAP2K1	544	PI4KB
105	RPS6KB1	545	EPHB4
106	AURKA	546	KLKB1
107	DPP7	547	PGK1
108	DPP8	548	EPHB3
109	DPP9	549	KDM5A
110	IGF1R	550	KDM5B
111	INSR	551	ABCG2
112	RET	552	PARP2
113	MET	553	NUAK1
114	NTRK2	554	MIF
115	ACVRL1	555	MPI
116	PIM3	556	CSNK1A1
117	ROS1	557	CSNK1D
118	NTRK3	558	PHOSPHO1
119	CA1	559	PPP5C
120	CA13	560	TBXAS1
121	CA5B	561	PDE4C
122	CA5A	562	CCNC
123	CA14	563	CSNK1E
124	CA4	564	BDKRB2
125	SLC22A12	565	GRM1
126	ADAM17	566	NR1I3
127	FLT3	567	TAAR1
128	CDK2	568	PIP4K2C
129	JAK3	569	GRM4
130	TYK2	570	KAT2B

131	MMP8	571	GABRB2
132	PGR	572	L3MBTL3
133	MMP13	573	TLR8
134	MMP9	574	HPGD
135	MMP14	575	GRIN2A
136	PSMB8	576	CHRNE
137	CNR1	577	HNMT
138	AR	578	HSD11B2
139	WEE1	579	ARHGDIA
140	PIK3CD	580	SCN4A
141	NPY5R	581	KCNMA1
142	ALOX5	582	NR1D1
143	CPB1	583	AOC1
144	PIK3CB	584	PHLPP2
145	PIK3C2B	585	MC1R
146	ESR2	586	MC3R
147	TNK2	587	HTR1E
148	PLA2G7	588	CHRNB1
149	CCNA1	589	CACNA1G
150	CCNA2	590	NOS3
151	KIF11	591	CHRNA1
152	CASR	592	CHRNG
153	PDE8B	593	CHRND
154	NOS2	594	FUCA1
155	CDK8	595	MAN1B1
156	ADORA1	596	MAN2A1
157	HDAC4	597	TYRO3
158	ALOX5AP	598	SELP
159	PRCP	599	MAP4K4
160	UTS2R	600	CDC25B
161	BACE1	601	GBA
162	EGLN1	602	MAP2K7
163	ATAD2	603	RELA
164	GHSR	604	MAP3K7
165	PRKCG	605	EHMT1
166	PRKCA	606	EHMT2
167	PRKCB	607	SLC5A2
168	PRKCH	608	CCKAR
169	SLC18A2	609	CCKBR
170	IDO1	610	NAT1
171	ABCB1	611	CTNNB1
172	MTAP	612	DUT
173	CSF1R	613	IL6ST
174	DUSP3	614	CACNA1C

175	HTR1F	615	GABRA6
176	TOP2A	616	GLP1R
177	PRKDC	617	PPOX
178	PNP	618	CETP
179	GRIN1	619	EDNRB
180	BAZ2B	620	PTGS1
181	BAZ2A	621	EDNRA
182	ALOX12	622	FBP1
183	MAPK9	623	PTGER1
184	FAP	624	MYLK2
185	MAPK11	625	ERBB4
186	KCNJ1	626	PRKD1
187	TERT	627	STK17B
188	KDM1A	628	EPHA5
189	EIF2AK3	629	ABL2
190	MMP3	630	EPHA8
191	MMP1	631	SLC2A1
192	MMP2	632	ATP6AP1
193	P2RX7	633	TRAP1
194	CHEK2	634	HSP90AB1
195	SLC6A2	635	VAV1
196	SLC6A4	636	PRKCE
197	OPRD1	637	NR1I2
198	OPRL1	638	PSMB9
199	SLC6A3	639	PSMB10
200	KCNH2	640	TKT
201	OPRK1	641	SIRT2
202	CHRNB3	642	CCR3
203	TNNC1	643	MKNK2
204	KISS1R	644	SCN1A
205	PLG	645	HCN4
206	MAOA	646	HCN1
207	CHRNA3	647	METAP1
208	CCNE2	648	EIF4A1
209	PDE9A	649	NMT1
210	PDE1C	650	PTPN11
211	NR3C2	651	SLC5A1
212	SERPINA6	652	BTK
213	SHBG	653	RASGRP3
214	SLC47A1	654	TBXA2R
215	SLC47A2	655	EP300
216	ADORA3	656	KNG1
217	CCNE1	657	KLK7
218	TNNI3	658	CASP9

219	TNNT2	659	ATR
220	CHRNA6	660	PTGER3
221	HTR3A	661	EED
222	CREBBP	662	SUZ12
223	ILK	663	EZH2
224	CXCR2	664	APP
225	NTRK1	665	CDC7
226	SPHK2	666	PDE4A
227	CXCR1	667	DBF4
228	SPHK1	668	PDK1
229	PDE3A	669	MAPK3
230	CHRNB4	670	PRSS1
231	CHRNA7	671	F7
232	CHRNA5	672	RXRA
233	HTR2A	673	ADAMTS5
234	CYP51A1	674	ADAMTS4
235	CHRNA2	675	FGR
236	PDE2A	676	EPHA3
237	FKBP1A	677	DDR2
238	TRPA1	678	DDR1
239	MDM2	679	BMP1
240	ADK	680	PPARG
241	HCRTR2	681	PPARA
242	HCRTR1	682	FCER2
243	MAPK8	683	MMP7
244	BDKRB1	684	CALCRL
245	PSEN1	685	TRPV1
246	ABCC9	686	MME
247	PER2	687	PDF
248	IRAK1	688	ECE1
249	AMPD2	689	HDAC8
250	CASP3	690	TK1
251	CLK3	691	MGLL
252	DYRK2	692	ABHD6
253	KCNK3	693	HDAC2
254	PGGT1B	694	HDAC5
255	CDK1	695	HDAC7
256	F2R	696	HDAC11
257	CRHR1	697	HDAC9
258	CFD	698	HDAC10
259	ITK	699	SLC10A2
260	CASP7	700	TK2
261	TNF	701	SLC6A5
262	NAAA	702	DGAT1

263	TNKS2	703	HPGDS
264	PIN1	704	TBK1
265	ALPL	705	ELOC
266	GSK3A	706	GRK2
267	ICMT	707	PLEC
268	TYMS	708	BUB1
269	AMPD3	709	P2RY12
270	WNT3A	710	ELOB
271	VDR	711	LIPA
272	GABRB3	712	PRMT3
273	ADORA2B	713	BCL2L1
274	VHL	714	MLX
275	BRPF1	715	ABCC1
276	PPP1CA	716	CSK
277	ROCK1	717	MC5R
278	GPR88	718	CHKA
279	GPR139	719	PLAT
280	CTSV	720	BIRC3
281	CASP8	721	CTSE
282	CYP11B1	722	INCENP
283	CYP19A1	723	IKBKE
284	PDE4D	724	CFTR
285	PREP	725	FGFR2
286	TACR3	726	GNRHR
287	MERTK	727	EBP
288	ADA	728	EBPL
289	C5AR1	729	GRK6
290	FNTA	730	CCR4
291	GABRG2	731	CASP6
292	GABRA5	732	RAPGEF4
293	GABRA1	733	GABRA4
294	CXCR3	734	BRAF
295	CTSC	735	AXL
296	BRD3	736	CSNK1G2
297	ROCK2	737	PLD1
298	GRK7	738	PLD2
299	TAOK2	739	PAK4
300	CDKL5	740	GPR142
301	ICK	741	POLR1A
302	PRPF4B	742	ACACB
303	PIP5K1C	743	CACNA1B
304	KIT	744	MRGPRX1
305	MAP2K6	745	MEN1
306	DYRK1A	746	MARK1

307	RPS6KA3	747	SAE1
308	AKT2	748	KCNQ3
309	DAPK3	749	CXCL8
310	PDPK1	750	GPBAR1
311	RPS6KA1	751	EPHX2
312	DAPK1	752	SCD
313	PRKCI	753	SLC16A1
314	CAMK2D	754	TRPC6
315	MAP3K9	755	TRPC3
316	PRKG2	756	AOC2
317	MAP2K4	757	PANK3
318	PRKACB	758	STK33
319	PKN2	759	PPIA
320	CDK7	760	GCGR
321	DAPK2	761	KCNJ5
322	RPS6KA4	762	KCNJ6
323	HCK	763	ELOVL6
324	CDK4	764	NR1H3
325	PKN1	765	KCNQ2
326	CSNK2A1	766	KCNJ3
327	FGFR1	767	UBA2
328	PLK4	768	CYP24A1
329	CAMK2G	769	CYP27B1
330	AAK1	770	GRM2
331	MARK2	771	KCNK9
332	RPS6KA2	772	VCP
333	AURKC	773	CMA1
334	STK10	774	C1R
335	PHKG1	775	MAP3K20
336	CSNK2A2	776	FRK
337	PRKACA	777	PTK6
338	CAMK2A	778	CIT
339	SLK	779	CDK19
340	CLK4	780	MAP3K19
341	CLK1	781	TNFRSF1A
342	CLK2	782	ALDH1A1
343	RPS6KA5	783	ALDH3A1
344	GAK	784	PTGFR
345	SRPK1	785	PTPN7
346	BMP2K	786	KCNA3
347	TNIK	787	CTRB1
348	HIPK2	788	KCNK2
349	HIPK3	789	H1F0
350	CDK16	790	KCNQ1

351	STK4	791	PLK3
352	STK3	792	KMT5A
353	MKNK1	793	CDC25C
354	AKT3	794	GJA1
355	MAP4K5	795	NDUFC2
356	PRKD2	796	SELE
357	RPS6KA6	797	HIF1A
358	MAP2K5	798	VEGFA
359	GRM5	799	NPPB
360	PYGL	800	KCNJ8
361	PSEN2	801	KCNJ12
362	MAPK10	802	KCNJ15
363	LIPE	803	SLC18A1
364	FPR1	804	ACE
365	FPR2	805	KCNJ10
366	CTSK	806	KCNJ14
367	CTSS	807	KCNH6
368	CCNB3	808	KCNH7
369	ELANE	809	TUBB3
370	MTNR1A	810	GSR
371	MTNR1B	811	GCDH
372	PTGES	812	ERO1B
373	BCL2A1	813	IVD
374	NQO2	814	DPYD
375	TDO2	815	TUBB
376	CCND3	816	TUBB4A
377	BRD2	817	POR
378	EIF4H	818	CYB5R1
379	PABPC1	819	TUBB6
380	P2RX3	820	FDXR
381	GSTO1	821	DAO
382	ENPP2	822	TXNRD1
383	HSD11B1	823	ACADS
384	SUMO1	824	CYB5R3
385	PSENEN	825	TUBB8
386	NCSTN	826	TUBB2B
387	APH1A	827	IL4I1
388	APH1B	828	AIFM1
389	CCND1	829	TUBB4B
390	CCND2	830	ACOX1
391	CCNB1	831	TUBB2A
392	CCNB2	832	ACADM
393	MAOB	833	XDH
394	ALK	834	ACAD8

395	DRD5	835	DLD
396	HRH2	836	GFER
397	HTR5A	837	ABCC2
398	PDGFRB	838	PRKAB1
399	MYLK	839	ACVR1
400	LCK	840	ASNA1
401	HTR7	841	ADRBK2
402	CCR1	842	ACSS2
403	SMO	843	NT5C2
404	TACR2	844	AMHR2
405	PTGS2	845	ASNS
406	SCARB1	846	MAP2
407	PSMB2	847	ADRBK1
408	PSMB1	848	IMPDH1
409	PSMB5	849	PRKAA1
410	CTSL	850	CREB1
411	OXTR	851	ACSS1
412	ADCY1	852	TUBA3C
413	HMGCR	853	MAPT
414	AVPR1A	854	AFG3L2
415	ACKR3	855	TUBA1C
416	CTSB	856	CDK15
417	CDK5R1	857	HINT1
418	CHRM2	858	ASS1
419	CCR5	859	TUBA1B
420	NCOR2	860	ACVR1B
421	LIMK1	861	ACSL1
422	PDE1B	862	ABCA1
423	FYN	863	ENPP1
424	PDGFRA	864	NAE1
425	YES1	865	PRKAB2
426	BLK	866	APAF1
427	CAMK1	867	TUBB1
428	PTK2	868	TUBA4A
429	MAP3K11	869	ABCC8
430	CDK5	870	ABCB11
431	HDAC3	871	MAP4
432	MPO	872	ABCG1
433	MCHR1	873	TUBA3D
434	HSP90AA1	874	ARAF
435	GALR1	875	TUBA1A
436	GALR2	876	SLC25A4
437	AVPR2	877	TUBA3E
438	AVPR1B	878	FKBP1B

439	PYGM	879	FGF2
440	BACE2		

Table S3. The targets related to excitotoxicity.

NO.	Targets	NO.	Targets
1	SLC1A2	388	IGKV2D-29
2	NOS1	389	MAP2K1
3	PRKN	390	CXCR4
4	GRIN1	391	TERT
5	MAPK8IP1	392	NFKB1
6	GRIN2B	393	PTEN
7	BDNF	394	PIK3R1
8	HTT	395	VIM
9	INPP4A	396	CACNA1S
10	PRR7	397	CAMK2A
11	GRIN2A	398	ADRB1
12	GRIA2	399	GSK3B
13	FOS	400	F2
14	APP	401	SLC16A1
15	GRIA1	402	LRRK2
16	SLC1A3	403	MAP2K3
17	RNF146	404	CACNA1A
18	DLG4	405	EEF2
19	RPS6KA5	406	MAPK9
20	SOD1	407	PLA2G6
21	FOLH1	408	TP63
22	JUN	409	YY1
23	NOS2	410	ACTN2
24	MAPK1	411	CACNA1D
25	NTRK2	412	ADORA1
26	BCL2	413	CYBB
27	CASP3	414	KEAP1
28	GDNF	415	DUSP1
29	PRKCG	416	GRM3
30	GRIN2D	417	GLP1R
31	GRM1	418	TFAP2A
32	PSEN1	419	PTGS1
33	CREB1	420	LEP
34	GRIN3A	421	HSPG2
35	CHAT	422	ANXA5
36	GRIN2C	423	CASP14
37	PIK3CG	424	CACNB4
38	PARP1	425	CD59
39	SGK1	426	HMGCR
40	TYRO3	427	GRM4
41	GPER1	428	SPP1
42	GAD1	429	SHMT2

43	APOE	430	OPRK1
44	NGF	431	PINK1
45	CNR1	432	MAP3K11
46	PVALB	433	PPARA
47	BLVRB	434	PIN1
48	SLC1A1	435	CACNA1F
49	TNF	436	ATP2A3
50	BAX	437	DKK1
51	FBXW7	438	CDK5R1
52	GRM5	439	EEF2K
53	CDK5	440	HSF1
54	CYCS	441	FOXO3
55	BCL2L1	442	SLC1A4
56	CNTF	443	SLC6A19
57	MAPK10	444	TRPM4
58	GAPDH	445	LAMC1
59	SLC8A1	446	NONO
60	MAPT	447	IL1A
61	GRIN3B	448	MDK
62	GFAP	449	TIMP1
63	TH	450	OPTN
64	SREBF1	451	PRDX3
65	NTF3	452	CACNB3
66	FGF2	453	CTSE
67	ACHE	454	CYSLTR1
68	OGDH	455	ACP1
69	HSPA4	456	ABCC5
70	GRINA	457	IL13
71	GRIA4	458	GLUD2
72	CAMK2G	459	GALR2
73	MAP2	460	TSPO
74	MTDH	461	MAP3K10
75	HDAC9	462	NDUFB8
76	NRTN	463	PRL
77	DLG3	464	NPY5R
78	PLAT	465	CHKA
79	CAPN1	466	CCK
80	LRP1	467	HIP1
81	CASP9	468	GCG
82	XDH	469	HPCA
83	GAS1	470	RPS27A
84	GLUL	471	SLC38A2
85	NTRK3	472	PAWR
86	PTGS2	473	NTSR1

87	AIFM1	474	ADGRL2
88	VEGFA	475	GPT
89	HSPA8	476	PDIA2
90	INS	477	NMNAT2
91	IL1R1	478	PPA1
92	GRIA3	479	NTS
93	SLC24A1	480	DPYSL3
94	SLC8A3	481	UBE2K
95	CYP19A1	482	ASIC2
96	CCL2	483	CD300LF
97	PIKFYVE	484	KCNIP3
98	VIP	485	VGF
99	GSR	486	NDOR1
100	NPY	487	NPY4R
101	SRR	488	SACS
102	DAO	489	AVEN
103	GRIK1	490	RIMKLA
104	ADORA2A	491	MYZAP
105	EPO	492	OR4D2
106	F5	493	ADPRS
107	FZR1	494	MIR107
108	IL1B	495	RNY5
109	AKT1	496	EGFR
110	DRD2	497	MTOR
111	NSF	498	PRKCD
112	SRC	499	SLC2A1
113	SNCA	500	MMP2
114	IGF1	501	CASP8
115	MAPK8	502	EGF
116	RYR1	503	STAT3
117	ADCYAP1	504	MYC
118	JAK2	505	PRKACA
119	KCNJ5	506	PRKAA2
120	GAD2	507	NT5E
121	TAT	508	ADA
122	CDKN3	509	CYP3A4
123	KIDINS220	510	BCHE
124	SIRT4	511	ADK
125	PLA2G2A	512	DPP4
126	CAST	513	ITGB1
127	ADCY10	514	DYRK1A
128	ITIH4	515	CTH
129	SST	516	FAS
130	TGFB1	517	F10

131	DPYSL2	518	RELA
132	SLC17A7	519	WNT5A
133	GRK2	520	NR3C1
134	TP53	521	PDGFB
135	PPARG	522	PLA2G7
136	CACNA1C	523	UCHL1
137	NTRK1	524	ARG1
138	OPRM1	525	CHRM2
139	XIAP	526	CAPN3
140	DNM1L	527	DAPK1
141	EPOR	528	ACTG1
142	IRS1	529	ADRB2
143	TGM2	530	GSK3A
144	BIRC3	531	KCNQ2
145	APAF1	532	EIF4E
146	CHRNA7	533	G6PD
147	SERPINI1	534	STXBP1
148	BIRC2	535	PSEN2
149	CX3CR1	536	PIK3CB
150	CS	537	MIF
151	IL9	538	PLG
152	GUK1	539	MAOA
153	FOSL2	540	PPP2CA
154	IL1RAPL2	541	PRKACB
155	CUL1	542	WNT7A
156	CREBBP	543	CASP2
157	FAAH	544	CASP6
158	HSP90AA1	545	CACNA2D1
159	SIRT1	546	CYP2D6
160	SPTAN1	547	B2M
161	F2R	548	EDN1
162	OPRL1	549	IFNG
163	TNFRSF1B	550	GABRA1
164	BIRC5	551	GLRB
165	DRD1	552	SLC6A4
166	GRM2	553	SLC12A1
167	SERPINF1	554	RIPK1
168	GLRX	555	PAK1
169	DLG1	556	P2RY12
170	LNX1	557	POMC
171	C9orf72	558	ATF6
172	DLG2	559	CYBA
173	GRP	560	DMD
174	TAX1BP3	561	CDKN1B

175	EFS	562	EDN3
176	ESR1	563	HTR2C
177	SMPD1	564	HTR3A
178	SLC12A2	565	ITPR2
179	MYD88	566	HIF1A
180	ODC1	567	HLA-A
181	TUBB3	568	IL10
182	CAPN2	569	GLRA1
183	KNG1	570	ENO1
184	ITGAM	571	GJB2
185	PRNP	572	KCNB1
186	OTC	573	HPRT1
187	KYNU	574	SPHK1
188	NMNAT1	575	PTGER3
189	SOX2	576	STX1A
190	UCP2	577	SLC25A12
191	APOD	578	SLC25A13
192	BCL2L2	579	SLC12A5
193	EGR1	580	MC1R
194	CX3CL1	581	MMP10
195	GAP43	582	NR3C2
196	IL17A	583	PC
197	SMN1	584	MBL2
198	KCNN1	585	PPP1CA
199	HCRT	586	PRKAA1
200	NUDT6	587	PRKACG
201	KARS1	588	HSPA9
202	FGFR3	589	PON1
203	FGFR1	590	WWOX
204	FGFR2	591	ABAT
205	ADAM10	592	CAMK4
206	PTPN11	593	ANGPT1
207	PPP3CA	594	DCTN1
208	IGF2	595	DCX
209	IDE	596	BCAT1
210	SDHB	597	BCAT2
211	LMNB1	598	ARHGEF2
212	MAP2K7	599	CYP1A2
213	TGFA	600	EIF4EBP1
214	SLIT2	601	EIF4G1
215	MAP2K4	602	HTR1D
216	COX5A	603	CRP
217	AGK	604	FXN
218	CDK20	605	KCNA1

219	NES	606	GAMT
220	MT3	607	PTGER4
221	MSBP1	608	SETD2
222	ADAM17	609	SLC5A6
223	SLC9A1	610	TGM1
224	ALOX5	611	VIPR1
225	TNFRSF1A	612	TKT
226	BACE1	613	SLC4A4
227	ENO2	614	PDE10A
228	SIGMAR1	615	PML
229	ADORA3	616	MC4R
230	CCNB1	617	NEFH
231	MST1	618	UBE2N
232	OPRD1	619	UBE2I
233	UBE2L3	620	VDAC1
234	APLNR	621	WFS1
235	DUSP3	622	WNT1
236	NTF4	623	ARG2
237	OPA1	624	BECN1
238	TNR	625	CALM1
239	JUNB	626	C1QB
240	ZFYVE9	627	CAPN5
241	SERPINE2	628	CYP2E1
242	ARTN	629	BAD
243	CXCL16	630	ADARB1
244	VRK3	631	AKAP9
245	LSM2	632	CYP2J2
246	HSPA14	633	FUS
247	PSPN	634	HTR1B
248	APLN	635	HTR4
249	LYN	636	KIF5B
250	CCNE1	637	KMO
251	GHSR	638	KCNN3
252	HCN2	639	KITLG
253	PRKCB	640	CSPG4
254	PARP2	641	HRH2
255	TNKS	642	S100B
256	CLN3	643	SP1
257	TNKS2	644	PXN
258	USF1	645	VAPB
259	CCNA1	646	TIMP3
260	S100A6	647	SUMO1
261	SKP1	648	SRD5A1
262	PARP4	649	PDE4A

263	RBX1	650	PTPN3
264	ACMSD	651	PPP2R2B
265	PARP3	652	PON2
266	TMEM147	653	PON3
267	CALB1	654	VAMP1
268	GLS	655	BAK1
269	SLC7A11	656	DGKZ
270	GRIK2	657	ATN1
271	FYN	658	AKR1A1
272	PTGER1	659	ALOX12
273	MAPK14	660	COX10
274	GLUD1	661	CALCA
275	PRKCA	662	S100A4
276	MEF2A	663	SLC18A3
277	NDUFS4	664	SOCS1
278	GCDH	665	SLC1A5
279	HSPA5	666	PDGFA
280	CALB2	667	PANX1
281	SLC1A6	668	UBE2D2
282	BCL2L11	669	UBE2G2
283	GRIK3	670	CAMK1
284	PICK1	671	CNP
285	SLC24A3	672	DDAH2
286	DUSP19	673	ATXN1
287	DNAH8	674	DNASE1
288	CAT	675	EEF1A1
289	CBS	676	CDKL5
290	ESR2	677	ARRB1
291	SLC2A3	678	GABARAPL2
292	MTHFR	679	GRIP1
293	ANXA2	680	HRH3
294	ATP7A	681	SP3
295	CNR2	682	TGM3
296	MEF2D	683	SOCS3
297	PDYN	684	SLC39A4
298	GAL	685	SNAP23
299	RET	686	MYOC
300	CTSB	687	NRXN3
301	PLA2G4A	688	MAP3K13
302	SCN1A	689	MC3R
303	P4HB	690	PPP1R1B
304	NGFR	691	PROCR
305	PARK7	692	VAMP2
306	BMP6	693	BAG1

307	CRH	694	AGRP
308	GRIK4	695	GABARAPL1
309	NPTX1	696	CREM
310	PTPA	697	FLOT1
311	MMP9	698	STX4
312	CTNNB1	699	RRAS
313	CASP1	700	PSMC4
314	MAPK3	701	SSTR5
315	TF	702	RHOT1
316	NFE2L2	703	SPTBN2
317	MEF2C	704	NDP
318	PTGER2	705	PTN
319	RYR2	706	TRAK1
320	GRM7	707	TRPM2
321	FGF1	708	CAPNS1
322	SCN2A	709	ATXN7
323	TARDBP	710	KIF5C
324	HMGB1	711	KLF13
325	SYP	712	EDN2
326	CHRNE	713	GOPC
327	ENDOG	714	STX1B
328	SLC1A7	715	PSMC6
329	RYR3	716	SLC17A6
330	PTPN5	717	NFIL3
331	CHPT1	718	MPP1
332	DPYSL4	719	PLEK
333	NAALAD2	720	MCF2
334	HMOX1	721	LGALS8
335	JAK3	722	NLRX1
336	NOS3	723	OLIG2
337	SLC6A3	724	AOC1
338	SOD2	725	CXCL8
339	MME	726	DBN1
340	APOA1	727	CRIPT
341	CASP7	728	MARCKS
342	CP	729	PARG
343	CACNA1B	730	BTG2
344	IL1RN	731	HAP1
345	ITPR1	732	SLC7A10
346	IL6	733	ZDHHC17
347	SNAP25	734	TLN2
348	TUBB	735	MPP2
349	HTR2A	736	MANF
350	HTR1A	737	NDRG2

351	GPHN	738	UNC5D
352	NCAM1	739	DBP
353	P2RX7	740	FIS1
354	PRKAB1	741	REG3A
355	AQP4	742	PLSCR3
356	CALM2	743	TPPP3
357	CAMKK2	744	ZNF326
358	CLU	745	CARD16
359	HRH1	746	RILPL1
360	ANG	747	SCT
361	ALS2	748	BCL2L15
362	CHRNB1	749	H1-2
363	B3GAT1	750	KYAT1
364	KCNN2	751	SLC38A6
365	GFRA1	752	RIMKLB
366	IDO1	753	MT-CO2
367	TRPM7	754	NECAB1
368	APLP2	755	H3-4
369	CACNB1	756	ICE2
370	GRIK5	757	MT-ND1
371	TACR2	758	POLR2M
372	MAOB	759	H1-1
373	QPRT	760	SELENOS
374	TAC1	761	UTP20
375	CIDEA	762	ATP5IF1
376	SETX	763	ATP5MF
377	LRRC8A	764	CCL3L1
378	NAIP	765	MIR21
379	AADAT	766	MIR132
380	ATCAY	767	MIR30A
381	LRRFIP1	768	LINC02141
382	NDNF	769	LOC110806262
383	NAALADL1	770	LOC108663993
384	PATJ	771	LOC108660406
385	GCOM1	772	LOC109504728
386	MIR142	773	REST
387	MT-RNR2	774	ANKK1

Table S4. The GO analysis of 214 intersective targets based on the P-value<0.01.

NO.	Category	Term	Count	P-value
1	BP	GO:0006468~protein phosphorylation	48	3.01E-30
2	BP	GO:0007268~chemical synaptic transmission	32	1.58E-23
3	BP	GO:0018105~peptidyl-serine phosphorylation	28	6.68E-23
4	BP	GO:0001666~response to hypoxia	27	1.36E-21
5	BP	GO:0018108~peptidyl-tyrosine phosphorylation	23	2.02E-19
6	BP	GO:0046777~protein autophosphorylation	25	3.13E-19
7	BP	GO:0060079~excitatory postsynaptic potential	17	8.45E-18
8	BP	GO:0007187~G-protein coupled receptor signaling pathway, coupled to cyclic nucleotide second messenger	17	1.11E-17
9	BP	GO:0007611~learning or memory	17	1.45E-17
10	BP	GO:0042493~response to drug	28	2.21E-17
11	BP	GO:0007568~aging	23	2.99E-16
12	BP	GO:0007165~signal transduction	52	3.71E-16
13	BP	GO:0043410~positive regulation of MAPK cascade	21	1.17E-15
14	BP	GO:0018107~peptidyl-threonine phosphorylation	16	1.41E-15
15	BP	GO:0035556~intracellular signal transduction	31	2.69E-15
16	BP	GO:0009410~response to xenobiotic stimulus	24	2.95E-15
17	BP	GO:0043525~positive regulation of neuron apoptotic process	15	6.21E-15
18	BP	GO:0070374~positive regulation of ERK1 and ERK2 cascade	23	9.86E-15
19	BP	GO:0010628~positive regulation of gene expression	32	1.33E-14
20	BP	GO:0006954~inflammatory response	29	1.45E-14
21	BP	GO:0019233~sensory perception of pain	14	2.48E-14
22	BP	GO:0043065~positive regulation of apoptotic process	26	3.27E-14
23	BP	GO:0045471~response to ethanol	18	1.17E-13
24	BP	GO:0032496~response to lipopolysaccharide	19	1.31E-13
25	BP	GO:0010629~negative regulation of gene expression	24	5.70E-13
26	BP	GO:0033138~positive regulation of peptidyl-serine phosphorylation	15	1.35E-12
27	BP	GO:0051402~neuron apoptotic process	14	1.49E-12
28	BP	GO:0043406~positive regulation of MAP kinase activity	14	4.22E-12
29	BP	GO:0001934~positive regulation of protein phosphorylation	19	1.98E-11

30	BP	GO:0007200~phospholipase C-activating G-protein coupled receptor signaling pathway	12	1.24E-10
31	BP	GO:0042752~regulation of circadian rhythm	12	1.48E-10
32	BP	GO:0051897~positive regulation of protein kinase B signaling	15	1.53E-10
33	BP	GO:0008542~visual learning	11	1.55E-10
34	BP	GO:0043536~positive regulation of blood vessel endothelial cell migration	11	1.55E-10
35	BP	GO:0006915~apoptotic process	29	1.69E-10
36	BP	GO:0043066~negative regulation of apoptotic process	27	1.83E-10
37	BP	GO:2000300~regulation of synaptic vesicle exocytosis	11	2.86E-10
38	BP	GO:0048661~positive regulation of smooth muscle cell proliferation	12	2.86E-10
39	BP	GO:0035094~response to nicotine	10	4.25E-10
40	BP	GO:0000187~activation of MAPK activity	12	4.57E-10
41	BP	GO:0071276~cellular response to cadmium ion	10	5.40E-10
42	BP	GO:0097190~apoptotic signaling pathway	12	7.15E-10
43	BP	GO:0048511~rhythmic process	12	8.26E-10
44	BP	GO:1901216~positive regulation of neuron death	10	8.52E-10
45	BP	GO:0000165~MAPK cascade	14	1.78E-09
46	BP	GO:0014068~positive regulation of phosphatidylinositol 3-kinase signaling	12	1.88E-09
47	BP	GO:1904646~cellular response to beta-amyloid	10	1.98E-09
48	BP	GO:0043524~negative regulation of neuron apoptotic process	15	2.52E-09
49	BP	GO:0051966~regulation of synaptic transmission, glutamatergic	9	2.79E-09
50	BP	GO:0071260~cellular response to mechanical stimulus	12	3.14E-09
51	BP	GO:0007613~memory	12	3.14E-09
52	BP	GO:0045821~positive regulation of glycolytic process	8	5.42E-09
53	BP	GO:0034614~cellular response to reactive oxygen species	9	5.76E-09
54	BP	GO:0045907~positive regulation of vasoconstriction	9	7.23E-09
55	BP	GO:0090398~cellular senescence	10	7.23E-09
56	BP	GO:0042220~response to cocaine	9	9.01E-09
57	BP	GO:0042981~regulation of apoptotic process	17	1.12E-08
58	BP	GO:0045776~negative regulation of blood pressure	8	2.33E-08

59	BP	GO:0006919~activation of cysteine-type endopeptidase activity involved in apoptotic process	11	2.37E-08
60	BP	GO:0007204~positive regulation of cytosolic calcium ion concentration	14	2.50E-08
61	BP	GO:0007169~transmembrane receptor protein tyrosine kinase signaling pathway	13	2.68E-08
62	BP	GO:0030335~positive regulation of cell migration	17	2.79E-08
63	BP	GO:0071222~cellular response to lipopolysaccharide	15	3.29E-08
64	BP	GO:0051968~positive regulation of synaptic transmission, glutamatergic	8	3.85E-08
65	BP	GO:0032355~response to estradiol	12	4.63E-08
66	BP	GO:0008284~positive regulation of cell proliferation	24	4.81E-08
67	BP	GO:0071456~cellular response to hypoxia	13	5.18E-08
68	BP	GO:0045429~positive regulation of nitric oxide biosynthetic process	9	6.00E-08
69	BP	GO:0048013~ephrin receptor signaling pathway	9	8.32E-08
70	BP	GO:0045893~positive regulation of transcription, DNA-templated	27	1.06E-07
71	BP	GO:0007189~adenylate cyclase-activating G-protein coupled receptor signaling pathway	12	2.16E-07
72	BP	GO:0097194~execution phase of apoptosis	7	2.54E-07
73	BP	GO:0045766~positive regulation of angiogenesis	13	2.94E-07
74	BP	GO:0007254~JNK cascade	9	3.07E-07
75	BP	GO:0050966~detection of mechanical stimulus involved in sensory perception of pain	6	4.34E-07
76	BP	GO:0042307~positive regulation of protein import into nucleus	8	4.97E-07
77	BP	GO:0009636~response to toxic substance	10	5.09E-07
78	BP	GO:1901215~negative regulation of neuron death	9	5.11E-07
79	BP	GO:0042177~negative regulation of protein catabolic process	8	5.83E-07
80	BP	GO:0042755~eating behavior	7	6.56E-07
81	BP	GO:0006508~proteolysis	19	7.36E-07
82	BP	GO:0030163~protein catabolic process	9	8.22E-07
83	BP	GO:0019722~calcium-mediated signaling	10	8.22E-07
84	BP	GO:0048143~astrocyte activation	6	8.77E-07
85	BP	GO:0001764~neuron migration	11	8.94E-07

86	BP	GO:1902895~positive regulation of pri-miRNA transcription from RNA polymerase II promoter	8	9.20E-07
87	BP	GO:0043154~negative regulation of cysteine-type endopeptidase activity involved in apoptotic process	9	9.21E-07
88	BP	GO:0038003~opioid receptor signaling pathway	5	9.84E-07
89	BP	GO:0043278~response to morphine	7	9.98E-07
90	BP	GO:0007623~circadian rhythm	9	1.03E-06
91	BP	GO:0045944~positive regulation of transcription from RNA polymerase II promoter	34	1.18E-06
92	BP	GO:0033674~positive regulation of kinase activity	9	1.43E-06
93	BP	GO:0071300~cellular response to retinoic acid	9	1.58E-06
94	BP	GO:0016310~phosphorylation	11	1.74E-06
95	BP	GO:0032091~negative regulation of protein binding	9	1.76E-06
96	BP	GO:0048167~regulation of synaptic plasticity	8	2.09E-06
97	BP	GO:0007193~adenylate cyclase-inhibiting G-protein coupled receptor signaling pathway	8	2.37E-06
98	BP	GO:1904707~positive regulation of vascular smooth muscle cell proliferation	8	2.37E-06
99	BP	GO:0071407~cellular response to organic cyclic compound	8	2.37E-06
100	BP	GO:0016241~regulation of macroautophagy	8	2.37E-06
101	BP	GO:0008285~negative regulation of cell proliferation	19	3.76E-06
102	BP	GO:0071230~cellular response to amino acid stimulus	8	3.85E-06
103	BP	GO:0007399~nervous system development	18	3.85E-06
104	BP	GO:0031663~lipopolysaccharide-mediated signaling pathway	7	4.11E-06
105	BP	GO:0038095~Fc-epsilon receptor signaling pathway	6	4.52E-06
106	BP	GO:0042593~glucose homeostasis	10	4.97E-06
107	BP	GO:0042311~vasodilation	7	5.56E-06
108	BP	GO:0007275~multicellular organism development	13	5.65E-06
109	BP	GO:0051090~regulation of sequence-specific DNA binding transcription factor activity	6	5.66E-06
110	BP	GO:1900273~positive regulation of long-term synaptic potentiation	6	5.66E-06

111	BP	GO:0007507~heart development	13	5.90E-06
112	BP	GO:1990090~cellular response to nerve growth factor stimulus	7	6.42E-06
113	BP	GO:0002931~response to ischemia	8	6.68E-06
114	BP	GO:0006816~calcium ion transport	9	7.00E-06
115	BP	GO:0051000~positive regulation of nitric-oxide synthase activity	6	8.60E-06
116	BP	GO:0060134~prepulse inhibition	5	9.62E-06
117	BP	GO:0043123~positive regulation of I-kappaB kinase/NF-kappaB signaling	12	9.94E-06
118	BP	GO:0098664~G-protein coupled serotonin receptor signaling pathway	6	1.26E-05
119	BP	GO:0031622~positive regulation of fever generation	4	1.31E-05
120	BP	GO:0014063~negative regulation of serotonin secretion	4	1.31E-05
121	BP	GO:0014042~positive regulation of neuron maturation	4	1.31E-05
122	BP	GO:0050728~negative regulation of inflammatory response	10	1.36E-05
123	BP	GO:0043627~response to estrogen	8	1.47E-05
124	BP	GO:0070301~cellular response to hydrogen peroxide	8	1.76E-05
125	BP	GO:0050900~leukocyte migration	6	1.80E-05
126	BP	GO:0048266~behavioral response to pain	5	1.81E-05
127	BP	GO:0048148~behavioral response to cocaine	5	1.81E-05
128	BP	GO:1904645~response to beta-amyloid	5	1.81E-05
129	BP	GO:0008630~intrinsic apoptotic signaling pathway in response to DNA damage	7	1.81E-05
130	BP	GO:0009314~response to radiation	6	2.12E-05
131	BP	GO:0097191~extrinsic apoptotic signaling pathway	7	2.27E-05
132	BP	GO:0050727~regulation of inflammatory response	9	2.34E-05
133	BP	GO:0019395~fatty acid oxidation	5	2.39E-05
134	BP	GO:0046677~response to antibiotic	6	2.49E-05
135	BP	GO:0045931~positive regulation of mitotic cell cycle	6	2.49E-05
136	BP	GO:0034605~cellular response to heat	7	2.54E-05
137	BP	GO:0042542~response to hydrogen peroxide	7	2.54E-05
138	BP	GO:0032229~negative regulation of synaptic transmission, GABAergic	4	2.60E-05
139	BP	GO:0051403~stress-activated MAPK cascade	5	3.09E-05

140	BP	GO:0007188~adenylate cyclase-modulating G-protein coupled receptor signaling pathway	7	3.15E-05
141	BP	GO:0016485~protein processing	8	3.19E-05
142	BP	GO:0007218~neuropeptide signaling pathway	9	3.27E-05
143	BP	GO:0051482~positive regulation of cytosolic calcium ion concentration involved in phospholipase C-activating G-protein coupled signaling pathway	6	3.39E-05
144	BP	GO:0035924~cellular response to vascular endothelial growth factor stimulus	6	3.39E-05
145	BP	GO:0051930~regulation of sensory perception of pain	6	3.39E-05
146	BP	GO:0050808~synapse organization	7	3.87E-05
147	BP	GO:0034612~response to tumor necrosis factor	6	3.91E-05
148	BP	GO:0051896~regulation of protein kinase B signaling	5	3.94E-05
149	BP	GO:0045987~positive regulation of smooth muscle contraction	5	3.94E-05
150	BP	GO:0051770~positive regulation of nitric-oxide synthase biosynthetic process	5	3.94E-05
151	BP	GO:0032880~regulation of protein localization	8	4.04E-05
152	BP	GO:0048678~response to axon injury	6	4.51E-05
153	BP	GO:0034121~regulation of toll-like receptor signaling pathway	4	4.52E-05
154	BP	GO:0007198~adenylate cyclase-inhibiting serotonin receptor signaling pathway	4	4.52E-05
155	BP	GO:0060440~trachea formation	4	4.52E-05
156	BP	GO:0002028~regulation of sodium ion transport	5	4.95E-05
157	BP	GO:0007626~locomotory behavior	8	5.07E-05
158	BP	GO:0007596~blood coagulation	8	5.46E-05
159	BP	GO:0007249~I-kappaB kinase/NF-kappaB signaling	7	5.71E-05
160	BP	GO:2001240~negative regulation of extrinsic apoptotic signaling pathway in absence of ligand	6	5.89E-05
161	BP	GO:0008625~extrinsic apoptotic signaling pathway via death domain receptors	6	6.70E-05
162	BP	GO:0030324~lung development	8	6.77E-05
163	BP	GO:0009612~response to mechanical stimulus	7	6.86E-05
164	BP	GO:0070212~protein poly-ADP-ribosylation	4	7.17E-05
165	BP	GO:0051092~positive regulation of NF-kappaB transcription factor activity	10	7.42E-05
166	BP	GO:0030168~platelet activation	7	7.50E-05

167	BP	GO:1902004~positive regulation of beta-amyloid formation	5	7.51E-05
168	BP	GO:0001659~temperature homeostasis	5	7.51E-05
169	BP	GO:0001975~response to amphetamine	6	7.59E-05
170	BP	GO:0032731~positive regulation of interleukin-1 beta production	7	8.19E-05
171	BP	GO:0031295~T cell costimulation	6	8.56E-05
172	BP	GO:0010976~positive regulation of neuron projection development	9	9.02E-05
173	BP	GO:0038083~peptidyl-tyrosine autophosphorylation	5	9.11E-05
174	BP	GO:0031175~neuron projection development	9	9.53E-05
175	BP	GO:0007196~adenylate cyclase-inhibiting G-protein coupled glutamate receptor signaling pathway	4	1.07E-04
176	BP	GO:2000641~regulation of early endosome to late endosome transport	4	1.07E-04
177	BP	GO:0051209~release of sequestered calcium ion into cytosol	6	1.08E-04
178	BP	GO:0035235~ionotropic glutamate receptor signaling pathway	5	1.09E-04
179	BP	GO:0006509~membrane protein ectodomain proteolysis	5	1.09E-04
180	BP	GO:0060252~positive regulation of glial cell proliferation	5	1.09E-04
181	BP	GO:0001525~angiogenesis	12	1.20E-04
182	BP	GO:0009611~response to wounding	7	1.24E-04
183	BP	GO:0045861~negative regulation of proteolysis	5	1.30E-04
184	BP	GO:0014065~phosphatidylinositol 3-kinase signaling	6	1.35E-04
185	BP	GO:0007186~G-protein coupled receptor signaling pathway	25	1.36E-04
186	BP	GO:0042391~regulation of membrane potential	8	1.39E-04
187	BP	GO:0001973~adenosine receptor signaling pathway	4	1.51E-04
188	BP	GO:0051901~positive regulation of mitochondrial depolarization	4	1.51E-04
189	BP	GO:0014074~response to purine-containing compound	4	1.51E-04
190	BP	GO:0060397~JAK-STAT cascade involved in growth hormone signaling pathway	4	1.51E-04
191	BP	GO:0034205~beta-amyloid formation	4	1.51E-04
192	BP	GO:0050796~regulation of insulin secretion	6	1.66E-04

193	BP	GO:0043393~regulation of protein binding	5	1.80E-04
194	BP	GO:0051924~regulation of calcium ion transport	5	1.80E-04
195	BP	GO:0010508~positive regulation of autophagy	7	1.82E-04
196	BP	GO:0048538~thymus development	6	1.84E-04
197	BP	GO:0032760~positive regulation of tumor necrosis factor production	8	1.99E-04
198	BP	GO:0007173~epidermal growth factor receptor signaling pathway	6	2.03E-04
199	BP	GO:0051967~negative regulation of synaptic transmission, glutamatergic	4	2.06E-04
200	BP	GO:0060020~Bergmann glial cell differentiation	4	2.06E-04
201	BP	GO:0030518~intracellular steroid hormone receptor signaling pathway	4	2.06E-04
202	BP	GO:1905564~positive regulation of vascular endothelial cell proliferation	5	2.09E-04
203	BP	GO:0046697~decidualization	5	2.09E-04
204	BP	GO:0016540~protein autoprocessing	5	2.09E-04
205	BP	GO:0032092~positive regulation of protein binding	7	2.11E-04
206	BP	GO:0032868~response to insulin	7	2.11E-04
207	BP	GO:0032869~cellular response to insulin stimulus	8	2.11E-04
208	BP	GO:0090263~positive regulation of canonical Wnt signaling pathway	8	2.11E-04
209	BP	GO:0031398~positive regulation of protein ubiquitination	7	2.26E-04
210	BP	GO:0048873~homeostasis of number of cells within a tissue	5	2.42E-04
211	BP	GO:0002052~positive regulation of neuroblast proliferation	5	2.42E-04
212	BP	GO:2001243~negative regulation of intrinsic apoptotic signaling pathway	5	2.42E-04
213	BP	GO:0050877~neurological system process	6	2.45E-04
214	BP	GO:0050890~cognition	6	2.45E-04
215	BP	GO:0001933~negative regulation of protein phosphorylation	7	2.60E-04
216	BP	GO:0008217~regulation of blood pressure	7	2.60E-04
217	BP	GO:0006974~cellular response to DNA damage stimulus	12	2.64E-04
218	BP	GO:0007600~sensory perception	4	2.73E-04
219	BP	GO:0070213~protein auto-ADP-ribosylation	4	2.73E-04

220	BP	GO:0042136~neurotransmitter biosynthetic process	4	2.73E-04
221	BP	GO:0043491~protein kinase B signaling	6	2.94E-04
222	BP	GO:0009408~response to heat	6	2.94E-04
223	BP	GO:0051602~response to electrical stimulus	5	3.18E-04
224	BP	GO:0043280~positive regulation of cysteine-type endopeptidase activity involved in apoptotic process	6	3.22E-04
225	BP	GO:0001932~regulation of protein phosphorylation	6	3.22E-04
226	BP	GO:0033209~tumor necrosis factor-mediated signaling pathway	6	3.51E-04
227	BP	GO:0031397~negative regulation of protein ubiquitination	6	3.51E-04
228	BP	GO:0048011~neurotrophin TRK receptor signaling pathway	4	3.52E-04
229	BP	GO:0050435~beta-amyloid metabolic process	4	3.52E-04
230	BP	GO:0010739~positive regulation of protein kinase A signaling	4	3.52E-04
231	BP	GO:0051091~positive regulation of sequence-specific DNA binding transcription factor activity	8	3.61E-04
232	BP	GO:2000463~positive regulation of excitatory postsynaptic potential	5	3.62E-04
233	BP	GO:2001235~positive regulation of apoptotic signaling pathway	5	3.62E-04
234	BP	GO:0043200~response to amino acid	5	3.62E-04
235	BP	GO:0042327~positive regulation of phosphorylation	5	3.62E-04
236	BP	GO:0001662~behavioral fear response	5	3.62E-04
237	BP	GO:0030592~DNA ADP-ribosylation	3	3.63E-04
238	BP	GO:0099553~trans-synaptic signaling by endocannabinoid, modulating synaptic transmission	3	3.63E-04
239	BP	GO:0031281~positive regulation of cyclase activity	3	3.63E-04
240	BP	GO:0021697~cerebellar cortex formation	3	3.63E-04
241	BP	GO:0071887~leukocyte apoptotic process	3	3.63E-04
242	BP	GO:0006979~response to oxidative stress	8	3.80E-04
243	BP	GO:0043552~positive regulation of phosphatidylinositol 3-kinase activity	5	4.10E-04
244	BP	GO:0042987~amyloid precursor protein catabolic process	4	4.44E-04
245	BP	GO:0000186~activation of MAPKK activity	4	4.44E-04

246	BP	GO:0008543~fibroblast growth factor receptor signaling pathway	6	4.50E-04
247	BP	GO:0007409~axonogenesis	7	4.62E-04
248	BP	GO:0040018~positive regulation of multicellular organism growth	5	4.63E-04
249	BP	GO:0043687~post-translational protein modification	5	4.63E-04
250	BP	GO:0060045~positive regulation of cardiac muscle cell proliferation	5	4.63E-04
251	BP	GO:0007190~activation of adenylate cyclase activity	5	4.63E-04
252	BP	GO:1900182~positive regulation of protein localization to nucleus	5	4.63E-04
253	BP	GO:0034644~cellular response to UV	6	4.87E-04
254	BP	GO:0032212~positive regulation of telomere maintenance via telomerase	5	5.20E-04
255	BP	GO:0051973~positive regulation of telomerase activity	5	5.20E-04
256	BP	GO:0016242~negative regulation of macroautophagy	4	5.50E-04
257	BP	GO:0003376~sphingosine-1-phosphate signaling pathway	4	5.50E-04
258	BP	GO:1900034~regulation of cellular response to heat	4	5.50E-04
259	BP	GO:1903827~regulation of cellular protein localization	4	5.50E-04
260	BP	GO:0030307~positive regulation of cell growth	7	5.52E-04
261	BP	GO:0019369~arachidonic acid metabolic process	5	5.82E-04
262	BP	GO:0090050~positive regulation of cell migration involved in sprouting angiogenesis	5	5.82E-04
263	BP	GO:0007612~learning	6	6.13E-04
264	BP	GO:0007166~cell surface receptor signaling pathway	12	6.13E-04
265	BP	GO:0050731~positive regulation of peptidyl-tyrosine phosphorylation	7	6.55E-04
266	BP	GO:0051146~striated muscle cell differentiation	4	6.72E-04
267	BP	GO:1903351~cellular response to dopamine	4	6.72E-04
268	BP	GO:1904019~epithelial cell apoptotic process	4	6.72E-04
269	BP	GO:0007216~G-protein coupled glutamate receptor signaling pathway	4	6.72E-04
270	BP	GO:1990044~protein localization to lipid particle	3	7.20E-04

271	BP	GO:0035585~calcium-mediated signaling using extracellular calcium source	3	7.20E-04
272	BP	GO:0014827~intestine smooth muscle contraction	3	7.20E-04
273	BP	GO:1990911~response to psychosocial stress	3	7.20E-04
274	BP	GO:0090170~regulation of Golgi inheritance	3	7.20E-04
275	BP	GO:0071549~cellular response to dexamethasone stimulus	5	7.22E-04
276	BP	GO:0010975~regulation of neuron projection development	5	7.22E-04
277	BP	GO:0046627~negative regulation of insulin receptor signaling pathway	5	8.00E-04
278	BP	GO:1904355~positive regulation of telomere capping	4	8.09E-04
279	BP	GO:0071380~cellular response to prostaglandin E stimulus	4	8.09E-04
280	BP	GO:2000310~regulation of NMDA receptor activity	4	8.09E-04
281	BP	GO:0051726~regulation of cell cycle	12	8.15E-04
282	BP	GO:0060291~long-term synaptic potentiation	6	8.75E-04
283	BP	GO:0035690~cellular response to drug	6	8.75E-04
284	BP	GO:0001890~placenta development	5	8.84E-04
285	BP	GO:0046326~positive regulation of glucose import	5	8.84E-04
286	BP	GO:0010595~positive regulation of endothelial cell migration	6	9.36E-04
287	BP	GO:0071880~adenylate cyclase-activating adrenergic receptor signaling pathway	4	9.63E-04
288	BP	GO:0042531~positive regulation of tyrosine phosphorylation of STAT protein	6	1.00E-03
289	BP	GO:0042060~wound healing	7	0.001001379
290	BP	GO:0032755~positive regulation of interleukin-6 production	7	0.001001379
291	BP	GO:1901224~positive regulation of NIK/NF-kappaB signaling	6	0.00106706
292	BP	GO:0030316~osteoclast differentiation	5	0.001069652
293	BP	GO:2000811~negative regulation of anoikis	4	0.001134577
294	BP	GO:0021954~central nervous system neuron development	4	0.001134577
295	BP	GO:2000573~positive regulation of DNA biosynthetic process	4	0.001134577
296	BP	GO:0097202~activation of cysteine-type endopeptidase activity	4	0.001134577
297	BP	GO:0010039~response to iron ion	4	0.001134577

298	BP	GO:0009409~response to cold	5	0.001172107
299	BP	GO:0071392~cellular response to estradiol stimulus	5	0.001172107
300	BP	GO:0032310~prostaglandin secretion	3	0.001191409
301	BP	GO:0014050~negative regulation of glutamate secretion	3	0.001191409
302	BP	GO:0045759~negative regulation of action potential	3	0.001191409
303	BP	GO:0072709~cellular response to sorbitol	3	0.001191409
304	BP	GO:1904000~positive regulation of eating behavior	3	0.001191409
305	BP	GO:0072734~cellular response to staurosporine	3	0.001191409
306	BP	GO:0070424~regulation of nucleotide-binding oligomerization domain containing signaling pathway	3	0.001191409
307	BP	GO:0045923~positive regulation of fatty acid metabolic process	3	0.001191409
308	BP	GO:0014061~regulation of norepinephrine secretion	3	0.001191409
309	BP	GO:0046889~positive regulation of lipid biosynthetic process	4	0.001323967
310	BP	GO:0050729~positive regulation of inflammatory response	7	0.001407426
311	BP	GO:0006874~cellular calcium ion homeostasis	7	0.001407426
312	BP	GO:0006366~transcription from RNA polymerase II promoter	10	0.001452617
313	BP	GO:0009725~response to hormone	5	0.001520469
314	BP	GO:0032722~positive regulation of chemokine production	5	0.001520469
315	BP	GO:0043407~negative regulation of MAP kinase activity	5	0.001520469
316	BP	GO:0009411~response to UV	5	0.001520469
317	BP	GO:0030154~cell differentiation	18	0.00152352
318	BP	GO:1902894~negative regulation of pri-miRNA transcription from RNA polymerase II promoter	4	0.001532104
319	BP	GO:0010288~response to lead ion	4	0.001532104
320	BP	GO:0045088~regulation of innate immune response	4	0.001532104
321	BP	GO:0031669~cellular response to nutrient levels	4	0.001532104
322	BP	GO:0032930~positive regulation of superoxide anion generation	4	0.001532104

323	BP	GO:0038096~Fc-gamma receptor signaling pathway involved in phagocytosis	4	0.001532104
324	BP	GO:0007584~response to nutrient	6	0.001544119
325	BP	GO:0001938~positive regulation of endothelial cell proliferation	6	0.001636728
326	BP	GO:0001837~epithelial to mesenchymal transition	5	0.001651013
327	BP	GO:0006809~nitric oxide biosynthetic process	4	0.001759638
328	BP	GO:0019222~regulation of metabolic process	4	0.001759638
329	BP	GO:0002223~stimulatory C-type lectin receptor signaling pathway	4	0.001759638
330	BP	GO:0019221~cytokine-mediated signaling pathway	8	0.001769671
331	BP	GO:1904428~negative regulation of tubulin deacetylation	3	0.001774091
332	BP	GO:0032025~response to cobalt ion	3	0.001774091
333	BP	GO:0034440~lipid oxidation	3	0.001774091
334	BP	GO:1904062~regulation of cation transmembrane transport	3	0.001774091
335	BP	GO:0007595~lactation	5	0.001789145
336	BP	GO:0032436~positive regulation of proteasomal ubiquitin-dependent protein catabolic process	6	0.002048351
337	BP	GO:0045737~positive regulation of cyclin-dependent protein serine/threonine kinase activity	5	0.002089071
338	BP	GO:0007005~mitochondrion organization	6	0.002162072
339	BP	GO:0051247~positive regulation of protein metabolic process	4	0.002275363
340	BP	GO:0006471~protein ADP-ribosylation	4	0.002275363
341	BP	GO:0038061~NIK/NF-kappaB signaling	4	0.002275363
342	BP	GO:0006527~arginine catabolic process	3	0.002465646
343	BP	GO:1903829~positive regulation of cellular protein localization	3	0.002465646
344	BP	GO:0007210~serotonin receptor signaling pathway	3	0.002465646
345	BP	GO:0036016~cellular response to interleukin-3	3	0.002465646
346	BP	GO:0014047~glutamate secretion	3	0.002465646
347	BP	GO:0032094~response to food	4	0.002564719
348	BP	GO:0071294~cellular response to zinc ion	4	0.002564719
349	BP	GO:0030225~macrophage differentiation	4	0.002564719
350	BP	GO:0060999~positive regulation of dendritic spine development	4	0.002564719

351	BP	GO:0007271~synaptic transmission, cholinergic	4	0.002564719
352	BP	GO:0048168~regulation of neuronal synaptic plasticity	4	0.002564719
353	BP	GO:0002862~negative regulation of inflammatory response to antigenic stimulus	4	0.002875801
354	BP	GO:0006936~muscle contraction	6	0.002943307
355	BP	GO:0010507~negative regulation of autophagy	5	0.00298721
356	BP	GO:0045600~positive regulation of fat cell differentiation	5	0.00298721
357	BP	GO:0034599~cellular response to oxidative stress	6	0.003243923
358	BP	GO:0030182~neuron differentiation	8	0.003263579
359	BP	GO:0048643~positive regulation of skeletal muscle tissue development	3	0.003263616
360	BP	GO:0071420~cellular response to histamine	3	0.003263616
361	BP	GO:0033129~positive regulation of histone phosphorylation	3	0.003263616
362	BP	GO:0051346~negative regulation of hydrolase activity	3	0.003263616
363	BP	GO:0010518~positive regulation of phospholipase activity	3	0.003263616
364	BP	GO:0002686~negative regulation of leukocyte migration	3	0.003263616
365	BP	GO:0035095~behavioral response to nicotine	3	0.003263616
366	BP	GO:0007197~adenylate cyclase-inhibiting G-protein coupled acetylcholine receptor signaling pathway	3	0.003263616
367	BP	GO:0014823~response to activity	5	0.003410339
368	BP	GO:1902042~negative regulation of extrinsic apoptotic signaling pathway via death domain receptors	4	0.003565188
369	BP	GO:0035066~positive regulation of histone acetylation	4	0.003565188
370	BP	GO:0070050~neuron cellular homeostasis	4	0.003565188
371	BP	GO:0048146~positive regulation of fibroblast proliferation	5	0.003636436
372	BP	GO:0031647~regulation of protein stability	6	0.003735435
373	BP	GO:0071363~cellular response to growth factor stimulus	5	0.003872495
374	BP	GO:0071280~cellular response to copper ion	4	0.003944449
375	BP	GO:0051928~positive regulation of calcium ion transport	4	0.003944449
376	BP	GO:0007257~activation of JUN kinase activity	4	0.003944449

377	BP	GO:0007205~protein kinase C-activating G-protein coupled receptor signaling pathway	4	0.003944449
378	BP	GO:0042420~dopamine catabolic process	3	0.004165584
379	BP	GO:0007220~Notch receptor processing	3	0.004165584
380	BP	GO:0034405~response to fluid shear stress	3	0.004165584
381	BP	GO:0071316~cellular response to nicotine	3	0.004165584
382	BP	GO:0071287~cellular response to manganese ion	3	0.004165584
383	BP	GO:0097553~calcium ion transmembrane import into cytosol	3	0.004165584
384	BP	GO:0001774~microglial cell activation	4	0.004347355
385	BP	GO:0014059~regulation of dopamine secretion	4	0.004347355
386	BP	GO:0048010~vascular endothelial growth factor receptor signaling pathway	4	0.004347355
387	BP	GO:0010800~positive regulation of peptidyl-threonine phosphorylation	4	0.004347355
388	BP	GO:0007631~feeding behavior	4	0.004347355
389	BP	GO:0022900~electron transport chain	5	0.004375312
390	BP	GO:0046330~positive regulation of JNK cascade	6	0.004669846
391	BP	GO:0043085~positive regulation of catalytic activity	4	0.004774325
392	BP	GO:0051881~regulation of mitochondrial membrane potential	4	0.004774325
393	BP	GO:0044346~fibroblast apoptotic process	3	0.005169175
394	BP	GO:0050795~regulation of behavior	3	0.005169175
395	BP	GO:0070244~negative regulation of thymocyte apoptotic process	3	0.005169175
396	BP	GO:0031000~response to caffeine	3	0.005169175
397	BP	GO:0001963~synaptic transmission, dopaminergic	3	0.005169175
398	BP	GO:2001056~positive regulation of cysteine-type endopeptidase activity	3	0.005169175
399	BP	GO:0032872~regulation of stress-activated MAPK cascade	3	0.005169175
400	BP	GO:0043267~negative regulation of potassium ion transport	3	0.005169175
401	BP	GO:0042053~regulation of dopamine metabolic process	3	0.005169175
402	BP	GO:0090647~modulation of age-related behavioral decline	3	0.005169175
403	BP	GO:0060259~regulation of feeding behavior	3	0.005169175
404	BP	GO:0043068~positive regulation of programmed cell death	3	0.005169175

405	BP	GO:0051272~positive regulation of cellular component movement	3	0.005169175
406	BP	GO:0010863~positive regulation of phospholipase C activity	3	0.005169175
407	BP	GO:0045860~positive regulation of protein kinase activity	5	0.005209249
408	BP	GO:0060078~regulation of postsynaptic membrane potential	4	0.005225756
409	BP	GO:0043029~T cell homeostasis	4	0.005225756
410	BP	GO:1900745~positive regulation of p38MAPK cascade	4	0.005225756
411	BP	GO:0032007~negative regulation of TOR signaling	4	0.005225756
412	BP	GO:0046718~viral entry into host cell	6	0.005304207
413	BP	GO:0019228~neuronal action potential	4	0.005702022
414	BP	GO:0048705~skeletal system morphogenesis	4	0.005702022
415	BP	GO:0007628~adult walking behavior	4	0.005702022
416	BP	GO:2001234~negative regulation of apoptotic signaling pathway	4	0.005702022
417	BP	GO:0032757~positive regulation of interleukin-8 production	5	0.005820589
418	BP	GO:0050804~modulation of synaptic transmission	5	0.005820589
419	BP	GO:0071466~cellular response to xenobiotic stimulus	5	0.005820589
420	BP	GO:0035249~synaptic transmission, glutamatergic	4	0.006203476
421	BP	GO:0016055~Wnt signaling pathway	8	0.006215834
422	BP	GO:1900015~regulation of cytokine production involved in inflammatory response	3	0.00627205
423	BP	GO:1903799~negative regulation of production of miRNAs involved in gene silencing by miRNA	3	0.00627205
424	BP	GO:0098976~excitatory chemical synaptic transmission	3	0.00627205
425	BP	GO:0071492~cellular response to UV-A	3	0.00627205
426	BP	GO:0071803~positive regulation of podosome assembly	3	0.00627205
427	BP	GO:0099527~postsynapse to nucleus signaling pathway	3	0.00627205
428	BP	GO:0097192~extrinsic apoptotic signaling pathway in absence of ligand	4	0.00673045

429	BP	GO:2000379~positive regulation of reactive oxygen species metabolic process	4	0.00673045
430	BP	GO:0051384~response to glucocorticoid	5	0.006824365
431	BP	GO:0070507~regulation of microtubule cytoskeleton organization	4	0.007283255
432	BP	GO:0007405~neuroblast proliferation	4	0.007283255
433	BP	GO:0061051~positive regulation of cell growth involved in cardiac muscle cell development	3	0.007471912
434	BP	GO:2000273~positive regulation of receptor activity	3	0.007471912
435	BP	GO:1990314~cellular response to insulin-like growth factor stimulus	3	0.007471912
436	BP	GO:0031284~positive regulation of guanylate cyclase activity	3	0.007471912
437	BP	GO:0042711~maternal behavior	3	0.007471912
438	BP	GO:0005979~regulation of glycogen biosynthetic process	3	0.007471912
439	BP	GO:0007416~synapse assembly	5	0.007553457
440	BP	GO:0046427~positive regulation of JAK-STAT cascade	4	0.007862184
441	BP	GO:0048709~oligodendrocyte differentiation	4	0.007862184
442	BP	GO:0009791~post-embryonic development	5	0.007936486
443	BP	GO:0007616~long-term memory	4	0.008467506
444	BP	GO:0008219~cell death	4	0.008467506
445	BP	GO:0048546~digestive tract morphogenesis	3	0.008766501
446	BP	GO:0045670~regulation of osteoclast differentiation	3	0.008766501
447	BP	GO:0060396~growth hormone receptor signaling pathway	3	0.008766501
448	BP	GO:0046902~regulation of mitochondrial membrane permeability	3	0.008766501
449	BP	GO:0051918~negative regulation of fibrinolysis	3	0.008766501
450	BP	GO:0033628~regulation of cell adhesion mediated by integrin	3	0.008766501
451	BP	GO:0033077~T cell differentiation in thymus	4	0.009099476
452	BP	GO:0070588~calcium ion transmembrane transport	6	0.00974396
453	BP	GO:0070555~response to interleukin-1	4	0.009758327
454	BP	GO:0042127~regulation of cell proliferation	7	0.009856978
1	CC	GO:0005886~plasma membrane	130	7.01E-29
2	CC	GO:0005887~integral component of plasma membrane	69	2.71E-26
3	CC	GO:0030425~dendrite	40	2.06E-24

4	CC	GO:0045121~membrane raft	30	4.76E-22
5	CC	GO:0043025~neuronal cell body	33	3.78E-19
6	CC	GO:0043005~neuron projection	32	4.55E-19
7	CC	GO:0045202~synapse	35	1.15E-18
8	CC	GO:0098978~glutamatergic synapse	31	2.83E-18
9	CC	GO:0099056~integral component of presynaptic membrane	17	5.61E-18
10	CC	GO:0030424~axon	29	1.04E-16
11	CC	GO:0005737~cytoplasm	113	3.16E-15
12	CC	GO:0099055~integral component of postsynaptic membrane	14	3.90E-15
13	CC	GO:0070161~anchoring junction	28	1.08E-12
14	CC	GO:0005829~cytosol	106	1.56E-12
15	CC	GO:0014069~postsynaptic density	20	5.34E-11
16	CC	GO:0005739~mitochondrion	45	7.08E-11
17	CC	GO:0009986~cell surface	28	6.50E-10
18	CC	GO:0043204~perikaryon	15	3.28E-09
19	CC	GO:0005901~caveola	11	7.19E-09
20	CC	GO:0031594~neuromuscular junction	11	8.18E-09
21	CC	GO:0043197~dendritic spine	14	2.38E-08
22	CC	GO:0048471~perinuclear region of cytoplasm	28	2.90E-08
23	CC	GO:0098793~presynapse	13	3.39E-08
24	CC	GO:0098794~postsynapse	12	3.95E-08
25	CC	GO:0016020~membrane	56	7.44E-08
26	CC	GO:0005634~nucleus	99	8.93E-08
27	CC	GO:0032991~macromolecular complex	26	9.00E-08
28	CC	GO:0005654~nucleoplasm	74	1.46E-07
29	CC	GO:0098685~Schaffer collateral - CA1 synapse	10	3.07E-07
30	CC	GO:0043235~receptor complex	14	4.75E-07
31	CC	GO:0045211~postsynaptic membrane	12	8.44E-07
32	CC	GO:0032839~dendrite cytoplasm	7	1.49E-06
33	CC	GO:0030426~growth cone	10	2.67E-05
34	CC	GO:0005741~mitochondrial outer membrane	11	3.28E-05
35	CC	GO:1904813~ficolin-1-rich granule lumen	9	4.72E-05
36	CC	GO:0044305~calyx of Held	5	5.93E-05
37	CC	GO:0098839~postsynaptic density membrane	6	7.23E-05
38	CC	GO:0043198~dendritic shaft	6	9.08E-05
39	CC	GO:0005769~early endosome	13	9.82E-05
40	CC	GO:0044294~dendritic growth cone	4	1.26E-04
41	CC	GO:0005641~nuclear envelope lumen	4	1.26E-04
42	CC	GO:0005925~focal adhesion	15	1.52E-04
43	CC	GO:0031588~nucleotide-activated protein kinase complex	4	1.72E-04
44	CC	GO:0016021~integral component of membrane	81	2.20E-04

45	CC	GO:0097060~synaptic membrane	5	4.14E-04
46	CC	GO:0098666~G-protein coupled serotonin receptor complex	3	6.38E-04
47	CC	GO:0043679~axon terminus	6	6.65E-04
48	CC	GO:0000307~cyclin-dependent protein kinase holoenzyme complex	5	7.76E-04
49	CC	GO:0005783~endoplasmic reticulum	25	8.43E-04
50	CC	GO:0005770~late endosome	8	9.27E-04
51	CC	GO:0005635~nuclear envelope	9	9.92E-04
52	CC	GO:0005789~endoplasmic reticulum membrane	23	0.001481
53	CC	GO:0016607~nuclear speck	13	0.001955
54	CC	GO:0043195~terminal bouton	5	0.002741
55	CC	GO:0005667~transcription factor complex	9	0.002968
56	CC	GO:0008021~synaptic vesicle	7	0.003142
57	CC	GO:0005943~phosphatidylinositol 3-kinase complex, class IA	3	0.003697
58	CC	GO:0017146~NMDA selective glutamate receptor complex	3	0.003697
59	CC	GO:0042734~presynaptic membrane	5	0.003741
60	CC	GO:0005911~cell-cell junction	8	0.004042
61	CC	GO:0000791~euchromatin	5	0.004202
62	CC	GO:0005788~endoplasmic reticulum lumen	10	0.004918
63	CC	GO:0005764~lysosome	10	0.005232
64	CC	GO:0031234~extrinsic component of cytoplasmic side of plasma membrane	5	0.005519
65	CC	GO:0099523~presynaptic cytosol	3	0.005571
66	CC	GO:0016324~apical plasma membrane	11	0.00595
67	CC	GO:0005768~endosome	10	0.006132
68	CC	GO:0030877~beta-catenin destruction complex	3	0.006639
69	CC	GO:1990909~Wnt signalosome	3	0.007793
70	CC	GO:0097449~astrocyte projection	3	0.00903
1	MF	GO:0004672~protein kinase activity	39	3.78E-25
2	MF	GO:0042802~identical protein binding	67	8.65E-20
3	MF	GO:0004674~protein serine/threonine kinase activity	33	1.39E-18
4	MF	GO:0016301~kinase activity	26	7.95E-18
5	MF	GO:0004713~protein tyrosine kinase activity	20	2.97E-17
6	MF	GO:0004714~transmembrane receptor protein tyrosine kinase activity	20	4.95E-17
7	MF	GO:0019899~enzyme binding	31	9.19E-17
8	MF	GO:0005524~ATP binding	59	9.20E-17
9	MF	GO:0030594~neurotransmitter receptor activity	16	2.43E-14

10	MF	GO:0005515~protein binding	188	8.53E-13
11	MF	GO:0051378~serotonin binding	8	1.63E-11
12	MF	GO:0004993~G-protein coupled serotonin receptor activity	10	1.06E-10
13	MF	GO:0019901~protein kinase binding	27	1.54E-10
14	MF	GO:0004708~MAP kinase kinase activity	8	6.19E-10
15	MF	GO:0042803~protein homodimerization activity	30	2.42E-09
16	MF	GO:0004707~MAP kinase activity	7	1.43E-08
17	MF	GO:0004712~protein serine/threonine/tyrosine kinase activity	8	2.73E-08
18	MF	GO:0048156~tau protein binding	9	3.56E-08
19	MF	GO:0097110~scaffold protein binding	10	8.13E-08
20	MF	GO:0005516~calmodulin binding	15	9.37E-08
21	MF	GO:0008233~peptidase activity	11	5.34E-07
22	MF	GO:0050660~flavin adenine dinucleotide binding	9	9.78E-07
23	MF	GO:0019903~protein phosphatase binding	10	1.09E-06
24	MF	GO:0020037~heme binding	12	1.14E-06
25	MF	GO:0046875~ephrin receptor binding	7	1.14E-06
26	MF	GO:0042923~neuropeptide binding	7	1.14E-06
27	MF	GO:0097200~cysteine-type endopeptidase activity involved in execution phase of apoptosis	5	1.93E-06
28	MF	GO:0004879~RNA polymerase II transcription factor activity, ligand-activated sequence-specific DNA binding	8	2.44E-06
29	MF	GO:0097153~cysteine-type endopeptidase activity involved in apoptotic process	5	3.18E-06
30	MF	GO:0050321~tau-protein kinase activity	6	4.00E-06
31	MF	GO:0044877~macromolecular complex binding	18	5.23E-06
32	MF	GO:0001540~beta-amyloid binding	9	5.90E-06
33	MF	GO:0004175~endopeptidase activity	9	6.43E-06
34	MF	GO:0043560~insulin receptor substrate binding	5	7.37E-06
35	MF	GO:0001223~transcription coactivator binding	7	9.68E-06
36	MF	GO:0042277~peptide binding	8	1.17E-05
37	MF	GO:0004715~non-membrane spanning protein tyrosine kinase activity	7	1.26E-05
38	MF	GO:0001609~G-protein coupled adenosine receptor activity	4	1.41E-05
39	MF	GO:0004985~opioid receptor activity	4	2.79E-05
40	MF	GO:0002020~protease binding	9	3.38E-05

41	MF	GO:0051721~protein phosphatase 2A binding	6	4.37E-05
42	MF	GO:0031625~ubiquitin protein ligase binding	14	4.71E-05
43	MF	GO:0004190~aspartic-type endopeptidase activity	6	7.47E-05
44	MF	GO:0004679~AMP-activated protein kinase activity	4	7.69E-05
45	MF	GO:0004683~calmodulin-dependent protein kinase activity	5	9.96E-05
46	MF	GO:0001640~adenylate cyclase inhibiting G-protein coupled glutamate receptor activity	4	1.14E-04
47	MF	GO:0030235~nitric-oxide synthase regulator activity	4	1.14E-04
48	MF	GO:0008022~protein C-terminus binding	11	1.34E-04
49	MF	GO:0008134~transcription factor binding	11	1.40E-04
50	MF	GO:0019900~kinase binding	8	1.61E-04
51	MF	GO:0097199~cysteine-type endopeptidase activity involved in apoptotic signaling pathway	4	1.62E-04
52	MF	GO:0070851~growth factor receptor binding	4	1.62E-04
53	MF	GO:0005102~receptor binding	15	1.77E-04
54	MF	GO:0005231~excitatory extracellular ligand-gated ion channel activity	6	1.85E-04
55	MF	GO:0004930~G-protein coupled receptor activity	22	2.05E-04
56	MF	GO:0061629~RNA polymerase II sequence-specific DNA binding transcription factor binding	10	3.00E-04
57	MF	GO:0016702~oxidoreductase activity, acting on single donors with incorporation of molecular oxygen, incorporation of two atoms of oxygen	4	3.76E-04
58	MF	GO:0004104~cholinesterase activity	3	3.80E-04
59	MF	GO:0004517~nitric-oxide synthase activity	3	3.80E-04
60	MF	GO:0004705~JUN kinase activity	3	3.80E-04
61	MF	GO:0015276~ligand-gated ion channel activity	5	4.48E-04
62	MF	GO:0004697~protein kinase C activity	4	4.75E-04
63	MF	GO:1904315~transmitter-gated ion channel activity involved in regulation of postsynaptic membrane potential	6	5.00E-04
64	MF	GO:0004698~calcium-dependent protein kinase C activity	4	7.19E-04
65	MF	GO:0008066~glutamate receptor activity	4	7.19E-04
66	MF	GO:0034617~tetrahydrobiopterin binding	3	7.54E-04
67	MF	GO:0043121~neurotrophin binding	3	7.54E-04

68	MF	GO:0090722~receptor-receptor interaction	3	7.54E-04
69	MF	GO:0005030~neurotrophin receptor activity	3	7.54E-04
70	MF	GO:0004197~cysteine-type endopeptidase activity	7	8.25E-04
71	MF	GO:1990404~protein ADP-ribosylase activity	4	8.66E-04
72	MF	GO:0051219~phosphoprotein binding	5	8.72E-04
73	MF	GO:0008234~cysteine-type peptidase activity	5	8.72E-04
74	MF	GO:0008201~heparin binding	9	9.07E-04
75	MF	GO:0022848~acetylcholine-gated cation-selective channel activity	4	0.00103
76	MF	GO:0044325~ion channel binding	8	0.00109
77	MF	GO:0005007~fibroblast growth factor-activated receptor activity	3	0.001248
78	MF	GO:0022849~glutamate-gated calcium ion channel activity	3	0.001248
79	MF	GO:0050661~NADP binding	5	0.001277
80	MF	GO:0031435~mitogen-activated protein kinase kinase kinase binding	4	0.001416
81	MF	GO:0005216~ion channel activity	5	0.001522
82	MF	GO:0030331~estrogen receptor binding	5	0.001656
83	MF	GO:0004969~histamine receptor activity	3	0.001858
84	MF	GO:0008131~primary amine oxidase activity	3	0.001858
85	MF	GO:0047485~protein N-terminus binding	7	0.002074
86	MF	GO:0003707~steroid hormone receptor activity	4	0.002145
87	MF	GO:0043548~phosphatidylinositol 3-kinase binding	4	0.002145
88	MF	GO:0008270~zinc ion binding	21	0.002238
89	MF	GO:0005158~insulin receptor binding	4	0.002431
90	MF	GO:0004709~MAP kinase kinase kinase activity	4	0.002431
91	MF	GO:0035240~dopamine binding	3	0.002581
92	MF	GO:0004972~NMDA glutamate receptor activity	3	0.003416
93	MF	GO:0004691~cAMP-dependent protein kinase activity	3	0.003416
94	MF	GO:0003950~NAD+ ADP-ribosyltransferase activity	4	0.003426
95	MF	GO:0034618~arginine binding	3	0.00436
96	MF	GO:0005496~steroid binding	4	0.004639
97	MF	GO:0051428~peptide hormone receptor binding	3	0.005409
98	MF	GO:0016491~oxidoreductase activity	9	0.006293
99	MF	GO:0008144~drug binding	3	0.006562
100	MF	GO:0042056~chemoattractant activity	4	0.007763

101	MF	GO:0042166~acetylcholine binding	3	0.007816
102	MF	GO:0009055~electron carrier activity	5	0.008184
103	MF	GO:0002039~p53 binding	5	0.008597
104	MF	GO:0016594~glycine binding	3	0.009169
105	MF	GO:0004706~JUN kinase kinase kinase activity	3	0.009169

Table S5. The KEGG pathway enrichment analysis of 214 intersective targets based on the P-value<0.01.

NO.	Category	Term	Count	Fold Enrichment	P-value
1	KEGG_PATHWAY	hsa05200: Pathways in cancer	64	4.748892	6.76E-27
2	KEGG_PATHWAY	hsa04020: Calcium signaling pathway	45	7.387681	2.21E-26
3	KEGG_PATHWAY	hsa04080: Neuroactive ligand-receptor interaction	51	5.550965	3.90E-24
4	KEGG_PATHWAY	hsa04024: cAMP signaling pathway	40	7.131397	9.41E-23
5	KEGG_PATHWAY	hsa04210: Apoptosis	31	8.981103	1.57E-20
6	KEGG_PATHWAY	hsa05161: Hepatitis B	33	8.026123	2.67E-20
7	KEGG_PATHWAY	hsa05417: Lipid and atherosclerosis	37	6.780631	3.11E-20
8	KEGG_PATHWAY	hsa04726: Serotonergic synapse	28	9.593279	2.60E-19
9	KEGG_PATHWAY	hsa05022: Pathways of neurodegeneration - multiple diseases	51	4.221532	8.15E-19
10	KEGG_PATHWAY	hsa04725: Cholinergic synapse	26	9.065709	2.76E-17
11	KEGG_PATHWAY	hsa05167: Kaposi sarcoma-associated herpesvirus infection	32	6.499128	6.54E-17
12	KEGG_PATHWAY	hsa04728: Dopaminergic synapse	27	8.059289	1.29E-16
13	KEGG_PATHWAY	hsa05010: Alzheimer disease	43	4.412087	1.90E-16
14	KEGG_PATHWAY	hsa04668: TNF signaling pathway	25	8.794859	2.70E-16
15	KEGG_PATHWAY	hsa05207: Chemical carcinogenesis - receptor activation	32	5.947316	9.71E-16
16	KEGG_PATHWAY	hsa04722: Neurotrophin signaling pathway	25	8.277514	1.28E-15
17	KEGG_PATHWAY	hsa04935: Growth hormone synthesis, secretion and action	25	8.277514	1.28E-15
18	KEGG_PATHWAY	hsa04750: Inflammatory mediator regulation of TRP channels	23	9.247166	1.89E-15
19	KEGG_PATHWAY	hsa01521: EGFR tyrosine kinase inhibitor resistance	21	10.47367	3.19E-15
20	KEGG_PATHWAY	hsa05145: Toxoplasmosis	24	8.443064	3.46E-15
21	KEGG_PATHWAY	hsa04926: Relaxin signaling pathway	25	7.635846	8.79E-15
22	KEGG_PATHWAY	hsa04071: Sphingolipid signaling pathway	24	7.946413	1.38E-14
23	KEGG_PATHWAY	hsa04931: Insulin resistance	23	8.390947	1.69E-14
24	KEGG_PATHWAY	hsa04066: HIF-1 signaling pathway	23	8.313965	2.06E-14
25	KEGG_PATHWAY	hsa05205: Proteoglycans in cancer	30	5.765995	2.21E-14
26	KEGG_PATHWAY	hsa04720: Long-term potentiation	19	11.17341	2.62E-14

27	KEGG_PATHWAY	hsa04933: AGE-RAGE signaling pathway in diabetic complications	22	8.668213	3.61E-14
28	KEGG_PATHWAY	hsa04010: MAPK signaling pathway	35	4.690591	4.70E-14
29	KEGG_PATHWAY	hsa05418: Fluid shear stress and atherosclerosis	25	7.086505	5.02E-14
30	KEGG_PATHWAY	hsa04912: GnRH signaling pathway	21	8.896992	9.36E-14
31	KEGG_PATHWAY	hsa04012: ErbB signaling pathway	20	9.270816	1.90E-13
32	KEGG_PATHWAY	hsa05215: Prostate cancer	21	8.530106	2.19E-13
33	KEGG_PATHWAY	hsa05163: Human cytomegalovirus infection	30	5.253462	2.58E-13
34	KEGG_PATHWAY	hsa04215: Apoptosis - multiple species	14	17.23792	2.66E-13
35	KEGG_PATHWAY	hsa01522: Endocrine resistance	21	8.443064	2.70E-13
36	KEGG_PATHWAY	hsa04664: Fc epsilon RI signaling pathway	18	10.42967	5.18E-13
37	KEGG_PATHWAY	hsa05031: Amphetamine addiction	18	10.27851	6.72E-13
38	KEGG_PATHWAY	hsa04540: Gap junction	19	8.507027	4.25E-12
39	KEGG_PATHWAY	hsa05030: Cocaine addiction	15	12.06152	8.58E-12
40	KEGG_PATHWAY	hsa04370: VEGF signaling pathway	16	10.68501	9.78E-12
41	KEGG_PATHWAY	hsa04917: Prolactin signaling pathway	17	9.568806	1.14E-11
42	KEGG_PATHWAY	hsa04915: Estrogen signaling pathway	22	6.281313	2.61E-11
43	KEGG_PATHWAY	hsa04014: Ras signaling pathway	28	4.694583	2.88E-11
44	KEGG_PATHWAY	hsa04921: Oxytocin signaling pathway	23	5.88456	3.09E-11
45	KEGG_PATHWAY	hsa04015: Rap1 signaling pathway	26	4.878215	7.65E-11
46	KEGG_PATHWAY	hsa05132: Salmonella infection	28	4.430631	1.11E-10
47	KEGG_PATHWAY	hsa04920: Adipocytokine signaling pathway	16	9.136456	1.11E-10
48	KEGG_PATHWAY	hsa05230: Central carbon metabolism in cancer	16	9.005935	1.38E-10
49	KEGG_PATHWAY	hsa04919: Thyroid hormone signaling pathway	20	6.512556	1.39E-10
50	KEGG_PATHWAY	hsa04151: PI3K-Akt signaling pathway	33	3.672971	2.18E-10
51	KEGG_PATHWAY	hsa04932: Non-alcoholic fatty liver disease	22	5.592395	2.47E-10
52	KEGG_PATHWAY	hsa05212: Pancreatic cancer	16	8.29494	4.77E-10

53	KEGG_PATHWAY	hsa05170: Human immunodeficiency virus 1 infection	25	4.64634	5.40E-10
54	KEGG_PATHWAY	hsa05152: Tuberculosis	23	5.034568	6.84E-10
55	KEGG_PATHWAY	hsa04510: Focal adhesion	24	4.704593	1.03E-09
56	KEGG_PATHWAY	hsa05415: Diabetic cardiomyopathy	24	4.658242	1.25E-09
57	KEGG_PATHWAY	hsa05166: Human T-cell leukemia virus 1 infection	25	4.437046	1.40E-09
58	KEGG_PATHWAY	hsa04713: Circadian entrainment	17	6.905324	2.00E-09
59	KEGG_PATHWAY	hsa04140: Autophagy - animal	20	5.58879	2.03E-09
60	KEGG_PATHWAY	hsa04936: Alcoholic liver disease	20	5.549432	2.29E-09
61	KEGG_PATHWAY	hsa04217: Necroptosis	21	5.203901	2.57E-09
62	KEGG_PATHWAY	hsa05210: Colorectal cancer	16	7.330412	2.93E-09
63	KEGG_PATHWAY	hsa05219: Bladder cancer	12	11.53199	3.33E-09
64	KEGG_PATHWAY	hsa05214: Glioma	15	7.880193	3.99E-09
65	KEGG_PATHWAY	hsa04068: FoxO signaling pathway	19	5.714644	4.01E-09
66	KEGG_PATHWAY	hsa05142: Chagas disease	17	6.566828	4.29E-09
67	KEGG_PATHWAY	hsa04723: Retrograde endocannabinoid signaling	20	5.324455	4.65E-09
68	KEGG_PATHWAY	hsa04072: Phospholipase D signaling pathway	20	5.324455	4.65E-09
69	KEGG_PATHWAY	hsa04620: Toll-like receptor signaling pathway	17	6.440543	5.74E-09
70	KEGG_PATHWAY	hsa04625:C-type lectin receptor signaling pathway	17	6.440543	5.74E-09
71	KEGG_PATHWAY	hsa05135: Yersinia infection	19	5.464368	8.31E-09
72	KEGG_PATHWAY	hsa04611: Platelet activation	18	5.719495	1.14E-08
73	KEGG_PATHWAY	hsa05131: Shigellosis	25	3.987952	1.19E-08
74	KEGG_PATHWAY	hsa04930: Type II diabetes mellitus	12	10.27851	1.26E-08
75	KEGG_PATHWAY	hsa04380: Osteoclast differentiation	18	5.540761	1.86E-08
76	KEGG_PATHWAY	hsa05231: Choline metabolism in cancer	16	6.432811	1.88E-08
77	KEGG_PATHWAY	hsa05130: Pathogenic Escherichia coli infection	22	4.400108	2.05E-08
78	KEGG_PATHWAY	hsa05223: Non-small cell lung cancer	14	7.661299	2.23E-08
79	KEGG_PATHWAY	hsa01524: Platinum drug resistance	14	7.55635	2.65E-08
80	KEGG_PATHWAY	hsa05169: Epstein-Barr virus infection	22	4.291194	3.18E-08

81	KEGG_PATHWAY	hsa05235: PD-L1 expression and PD-1 checkpoint pathway in cancer	15	6.640612	3.98E-08
82	KEGG_PATHWAY	hsa05164: Influenza A	20	4.6083	5.13E-08
83	KEGG_PATHWAY	hsa05222: Small cell lung cancer	15	6.424071	6.14E-08
84	KEGG_PATHWAY	hsa04062: Chemokine signaling pathway	21	4.309481	6.71E-08
85	KEGG_PATHWAY	hsa04657:IL-17 signaling pathway	15	6.287388	8.13E-08
86	KEGG_PATHWAY	hsa05020: Prion disease	25	3.608147	8.28E-08
87	KEGG_PATHWAY	hsa05120: Epithelial cell signaling in Helicobacter pylori infection	13	7.317322	1.44E-07
88	KEGG_PATHWAY	hsa04724: Glutamatergic synapse	16	5.52996	1.50E-07
89	KEGG_PATHWAY	hsa04621: NOD-like receptor signaling pathway	20	4.282714	1.67E-07
90	KEGG_PATHWAY	hsa04916: Melanogenesis	15	5.851629	2.05E-07
91	KEGG_PATHWAY	hsa04914: Progesterone-mediated oocyte maturation	15	5.79426	2.32E-07
92	KEGG_PATHWAY	hsa04660: T cell receptor signaling pathway	15	5.682832	2.97E-07
93	KEGG_PATHWAY	hsa04910: Insulin signaling pathway	17	4.889171	3.07E-07
94	KEGG_PATHWAY	hsa05133: Pertussis	13	6.739639	3.67E-07
95	KEGG_PATHWAY	hsa04371: Apelin signaling pathway	17	4.818823	3.75E-07
96	KEGG_PATHWAY	hsa05162: Measles	17	4.818823	3.75E-07
97	KEGG_PATHWAY	hsa05160: Hepatitis C	18	4.517308	3.89E-07
98	KEGG_PATHWAY	hsa05032: Morphine addiction	14	6.061687	3.95E-07
99	KEGG_PATHWAY	hsa04929: GnRH secretion	12	7.387681	4.70E-07
100	KEGG_PATHWAY	hsa05208: Chemical carcinogenesis - reactive oxygen species	21	3.710405	7.77E-07
101	KEGG_PATHWAY	hsa05034: Alcoholism	19	4.003307	1.02E-06
102	KEGG_PATHWAY	hsa04261: Adrenergic signaling in cardiomyocytes	17	4.465443	1.06E-06
103	KEGG_PATHWAY	hsa04923: Regulation of lipolysis in adipocytes	11	7.739476	1.10E-06
104	KEGG_PATHWAY	hsa04218: Cellular senescence	17	4.293695	1.80E-06
105	KEGG_PATHWAY	hsa04211: Longevity regulating pathway	13	5.755197	2.10E-06
106	KEGG_PATHWAY	hsa05165: Human papillomavirus infection	25	2.975904	2.83E-06
107	KEGG_PATHWAY	hsa04550: Signaling pathways regulating pluripotency of stem cells	16	4.4085	2.89E-06

108	KEGG_PATHWAY	hsa04960: Aldosterone-regulated sodium reabsorption	9	9.584019	2.97E-06
109	KEGG_PATHWAY	hsa05171: Coronavirus disease - COVID-19	20	3.396635	5.76E-06
110	KEGG_PATHWAY	hsa05211: Renal cell carcinoma	11	6.281313	7.87E-06
111	KEGG_PATHWAY	hsa04150: mTOR signaling pathway	16	4.041125	8.51E-06
112	KEGG_PATHWAY	hsa04064:NF-kappa B signaling pathway	13	4.925121	1.10E-05
113	KEGG_PATHWAY	hsa05012: Parkinson disease	21	3.110603	1.18E-05
114	KEGG_PATHWAY	hsa05213: Endometrial cancer	10	6.79327	1.28E-05
115	KEGG_PATHWAY	hsa04928: Parathyroid hormone synthesis, secretion and action	13	4.832194	1.34E-05
116	KEGG_PATHWAY	hsa05203: Viral carcinogenesis	18	3.476556	1.45E-05
117	KEGG_PATHWAY	hsa04659: Th17 cell differentiation	13	4.742709	1.62E-05
118	KEGG_PATHWAY	hsa05225: Hepatocellular carcinoma	16	3.752473	2.08E-05
119	KEGG_PATHWAY	hsa05226: Gastric cancer	15	3.96654	2.23E-05
120	KEGG_PATHWAY	hsa04666: Fc gamma R-mediated phagocytosis	12	4.874346	3.04E-05
121	KEGG_PATHWAY	hsa04925: Aldosterone synthesis and secretion	12	4.824608	3.35E-05
122	KEGG_PATHWAY	hsa05221: Acute myeloid leukemia	10	5.880741	4.22E-05
123	KEGG_PATHWAY	hsa05146: Amoebiasis	12	4.635408	4.87E-05
124	KEGG_PATHWAY	hsa04911: Insulin secretion	11	5.039658	5.64E-05
125	KEGG_PATHWAY	hsa05017: Spinocerebellar ataxia	14	3.857437	6.21E-05
126	KEGG_PATHWAY	hsa04922: Glucagon signaling pathway	12	4.4188	7.59E-05
127	KEGG_PATHWAY	hsa04022: cGMP-PKG signaling pathway	15	3.539009	7.91E-05
128	KEGG_PATHWAY	hsa05134: Legionellosis	9	6.221205	8.25E-05
129	KEGG_PATHWAY	hsa05224: Breast cancer	14	3.752473	8.27E-05
130	KEGG_PATHWAY	hsa05206: MicroRNAs in cancer	21	2.669098	1.05E-04
131	KEGG_PATHWAY	hsa04730: Long-term depression	9	5.910145	1.20E-04
132	KEGG_PATHWAY	hsa04213: Longevity regulating pathway - multiple species	9	5.719495	1.51E-04
133	KEGG_PATHWAY	hsa04662: B cell receptor signaling pathway	10	4.804996	2.09E-04
134	KEGG_PATHWAY	hsa05216: Thyroid cancer	7	7.454237	2.92E-04
135	KEGG_PATHWAY	hsa04742: Taste transduction	10	4.581508	3.00E-04
136	KEGG_PATHWAY	hsa04613: Neutrophil extracellular trap formation	15	3.110603	3.10E-04
137	KEGG_PATHWAY	hsa04650: Natural killer cell mediated cytotoxicity	12	3.752473	3.27E-04

138	KEGG_PATHWAY	hsa04310: Wnt signaling pathway	14	3.244785	3.55E-04
139	KEGG_PATHWAY	hsa04622: RIG-I-like receptor signaling pathway	9	5.065839	3.56E-04
140	KEGG_PATHWAY	hsa04810: Regulation of actin cytoskeleton	16	2.891814	3.94E-04
141	KEGG_PATHWAY	hsa05218: Melanoma	9	4.925121	4.32E-04
142	KEGG_PATHWAY	hsa04934: Cushing syndrome	13	3.304597	5.34E-04
143	KEGG_PATHWAY	hsa04270: Vascular smooth muscle contraction	12	3.528445	5.56E-04
144	KEGG_PATHWAY	hsa05220: Chronic myeloid leukemia	9	4.665904	6.25E-04
145	KEGG_PATHWAY	hsa05140: Leishmaniasis	9	4.605308	6.82E-04
146	KEGG_PATHWAY	hsa04152: AMPK signaling pathway	11	3.611755	8.79E-04
147	KEGG_PATHWAY	hsa00330: Arginine and proline metabolism	7	5.516135	0.001518
148	KEGG_PATHWAY	hsa04924: Renin secretion	8	4.568228	0.001696
149	KEGG_PATHWAY	hsa04114: Oocyte meiosis	11	3.308478	0.001718
150	KEGG_PATHWAY	hsa04727: GABAergic synapse	9	3.984367	0.001769
151	KEGG_PATHWAY	hsa05016: Huntington disease	18	2.317704	0.001854
152	KEGG_PATHWAY	hsa04520: Adherens junction	8	4.439545	0.002003
153	KEGG_PATHWAY	hsa04658: Th1 and Th2 cell differentiation	9	3.854442	0.002186
154	KEGG_PATHWAY	hsa05143: African trypanosomiasis	6	6.389346	0.00221
155	KEGG_PATHWAY	hsa04670: Leukocyte transendothelial migration	10	3.456225	0.002304
156	KEGG_PATHWAY	hsa04115: p53 signaling pathway	8	4.317914	0.002353
157	KEGG_PATHWAY	hsa04630: JAK-STAT signaling pathway	12	2.91859	0.002613
158	KEGG_PATHWAY	hsa04971: Gastric acid secretion	8	4.14747	0.002962
159	KEGG_PATHWAY	hsa05033: Nicotine addiction	6	5.910145	0.003138
160	KEGG_PATHWAY	hsa00380: Tryptophan metabolism	6	5.628709	0.003895
161	KEGG_PATHWAY	hsa05014: Amyotrophic lateral sclerosis	19	2.056644	0.004824
162	KEGG_PATHWAY	hsa04360: Axon guidance	12	2.597866	0.006332
163	KEGG_PATHWAY	hsa04970: Salivary secretion	8	3.426171	0.00847
164	KEGG_PATHWAY	hsa04137: Mitophagy - animal	7	3.830649	0.00945

Table S6. Acute toxicity of humantenirien in ICR male and female mice.

Male (n=5)			Female (n=5)		
Group	Dose ($mg \cdot kg^{-1}$)	Death	Group	Dose ($mg \cdot kg^{-1}$)	Death
1	0.1	0	1	0.045	0
2	0.12	0	2	0.056	1
3	0.13	1	3	0.069	3
4	0.16	3	4	0.086	3
5	0.18	5	5	0.11	5

Table S7. The LD_{50} of *Gelsemium* alkaloids in mice.

**Compounds**	**Animals**	**Route**	**LD_{50} (mg/kg)**	**Reference**
19α-Hydroxygelsamydine	-	-	-	-
Gelseiridone	-	-	-	-
14-Dehydroxygelsefuranidine	-	-	-	-
Humantenirine	Mice	i.p.	0.149	-
Gelsenicine	Mice	i.p.	0.185	[1]
14-Hydroxygelsenicine	Mice	p.o.	0.295	[2]
Humantendine	Mice	i.p.	0.21	[3]
Gelsemine	Mice	i.p.	56.2	[4]
Koumine	Mice	s.c.	99	[4]
Koumidine	Mice	i.p.	>125	[4]

References

1. Du, X.B., Y.H. Dai, C.L. Zhang, et al., *Studies on the Gelsemium Alkaloids—— I . The structure of Gelsenicine* Acta Chim. Sinica, 1982(12) p.(1137-1141).
2. Yang, S., Y. Liu, F. Sun, et al., *Gelsedine-type alkaloids: Discovery of natural neurotoxins presented in toxic honey*. J. Hazard. Mater., 2020. **381** p.(120999). DOI: 10.1016/j.jhazmat.2019.120999.
3. Zhou, Y.P., W. Xu, and X.Y. Chen, *Toxicity and respiratory inhibition of humantemine*. Chin. J. Pharmacol. Toxicol., 1995. **9**(01) p.(69-72).
4. Jin, G.L., Y.P. Su, M. Liu, et al., *Medicinal plants of the genus Gelsemium (Gelsemiaceae, Gentianales)--a review of their phytochemistry, pharmacology, toxicology and traditional use*. J. Ethnopharmacol., 2014. **152**(1) p.(33-52). DOI: 10.1016/j.jep.2014.01.003.
